# Redundant Glycerol‐3‐Phosphate Acyltransferases Regulate Thermo‐Sensitive Genic Male Sterility in Rice

**DOI:** 10.1111/pbi.70649

**Published:** 2026-04-01

**Authors:** Siqi Cheng, Hai Zheng, Yunlu Tian, Chaolong Wang, Xiaowen Yu, Kun Dong, Jian Wang, Jiayu Lu, Keyi Chen, Xiaodong He, Kun Shao, Junwen Gao, Bowen Yao, Yang Hu, Zhenwei Xie, Ling He, Zhao Xie, Yuantao Zhu, Song Guo, Dekun Lei, Anqi Jian, Xufei Zhu, Hao Yu, Shihao Zhang, Minrui Chen, Yun Chen, Ling Jiang, Yulong Ren, Xiuping Guo, Shijia Liu, Xi Liu, Shanshan Zhu, Zhigang Zhao, Jianmin Wan

**Affiliations:** ^1^ State Key Laboratory for Crop Genetics & Germplasm Enhancement and Utilization, Nanjing Agricultural University, Zhongshan Biological Breeding Laboratory Nanjing China; ^2^ State Key Laboratory of Crop Gene Resources and Breeding, National Key Facility for Crop Gene Resources and Genetic Improvement, Institute of Crop Sciences, Chinese Academy of Agricultural Sciences Beijing China

**Keywords:** GPAT, hybrid breeding, lipid metabolism, rice, TGMS

## Abstract

Thermo‐sensitive genic male sterility (TGMS) lines play a pivotal role in two‐line hybrid rice breeding. However, the availability of elite TGMS germplasm remains limited, and the molecular mechanisms underlying fertility transition of TGMS lines are still poorly understood. Here, we identified a novel TGMS line, *Osgpat6.1*, which exhibits temperature‐dependent fertility transition, with male sterility under high‐temperature (HT) and restored fertility under low‐temperature (LT). Cytological observations revealed that the TGMS line exhibits disrupted programmed cell death (PCD) of the tapetum under HT conditions. This disruption leads to delayed tapetum degradation and defective pollen exine formation. Map‐based cloning identified a premature termination mutation in glycerol‐3‐phosphate acyltransferase 6.1 (*OsGPAT6.1*), which is highly expressed in anthers and essential for lipid biosynthesis. Lipidomic profiling revealed that disruption of *OsGPAT6.1* perturbed lipid metabolism during pollen development. Genetic studies further revealed that male sterility in *Osgpat6.1* mutant was restored through functional redundancy with its homologues *OsGPAT6.2* and *OsGPAT6.3* under LT. Overexpression of *OsGPAT6.2* or *OsGPAT6.3* partially restored the fertility of *Osgpat6.1* mutant under HT. However, simultaneous disruption of all three homologues abolished fertility restoration even under LT. Notably, introgression of the *Osgpat6.1* allele into diverse rice cultivars produced stable TGMS lines without compromising hybrid vigor. Collectively, these findings demonstrate that dysfunction of GPAT confers TGMS in rice by disrupting glycerolipid metabolism and provides TGMS germplasm to facilitate two‐line hybrid rice breeding.

## 1 Introduction

Male sterility (MS) is a widespread natural phenomenon that serves a crucial function in hybrid breeding systems, especially in self‐pollinating crops such as rice (Han et al. 2023; Ouyang and Zhang 2018). Natural MS systems are primarily classified into cytoplasmic male sterility (CMS) and genic male sterility (GMS) (Cai et al. 2024; Vasupalli et al. 2025). These two systems play distinct roles in hybrid breeding: CMS serves as the foundation of the three‐line breeding system, whereas GMS, particularly photo/thermo‐sensitive genic male sterility (P/TGMS), forms the basis of the more advanced two‐line hybrid breeding system (Fan and Zhang 2017; Ma 2005; Vasupalli et al. 2025). P/TGMS lines exhibit environment‐dependent fertility transitions, where MS is induced under long‐day/high‐temperature (LD/HT) conditions, while fertility is restored under short‐day/low‐temperature (SD/LT) conditions (Wang et al. 2026). This sensitivity offers significant advantages in hybrid breeding programs, including simplified seed production procedures, reduced operational costs, enhanced breeding efficiency, and expanded germplasm utilization. Empirical evidence indicates that the two‐line breeding system can increase crop yields by 5%–10% compared to the three‐line system (Zhang et al. 2022). As a result, P/TGMS has been increasingly adopted in modern breeding programs. Ongoing research and development in P/TGMS technology are essential to further enhance crop productivity and support global food security.

Key genes such as *PMS1*, *PMS3*, and *TMS5*, which are widely utilized in two‐line hybrid breeding, regulate P/TGMS through roles in small RNA and ribonuclease Z (RNase Z) function (Ding et al. 2012; Fan et al. 2016; Liu et al. 2025; Peng et al. 2021, 2023; Yan et al. 2024; Zhou et al. 2025, 2012, 2014). Recent studies have revealed additional regulatory mechanisms underlying P/TGMS. For instance, delayed pollen development under LT has been proposed as a conserved mechanism for P/TGMS in both Arabidopsis and rice (Han et al. 2023; Peng et al. 2022; Wang et al. 2026, 2025, 2024; Zhang et al. 2020, 2022; Zhou et al. 2024; Zhu, Lou, et al. 2020). This is supported by genetic evidence showing that fertility can be restored in male‐sterile mutants under HT or LD conditions through artificially delaying pollen development (Zhang et al. 2020; Zhu, Lou, et al. 2020). Furthermore, the R2R3 MYB transcription factors CSA and CSA2 coordinately regulate photo‐sensitive male fertility in rice by controlling sugar transport pathways in a light‐dependent manner (Wang et al. 2021; Zhang et al. 2012). UGP1 contributes to TGMS in rice by undergoing temperature‐dependent intron retention, which suppresses protein accumulation and disrupts fertility under HT (Chen et al. 2007). *OsMS1*, which encodes a PHD‐finger protein, regulates tapetum PCD via protein–protein interactions, and its natural allele *OsMS1* ^*wenmin1*^ exhibits impaired nuclear localization and downstream gene activation under HT, resulting in pollen abortion (Wu et al. 2022). Gene functional redundancy is also recognized as a potential factor in regulating P/TGMS, as evidenced by the failure of LT to restore fertility in double mutants of *TMS10* and *TMS10‐like* compared to single mutations (Yu et al. 2017). Despite these advances, the regulatory mechanisms of P/TGMS remain to be elucidated.

Lipid metabolism is a fundamental biological process essential for energy storage and cellular function. Glycerol‐3‐phosphate acyltransferase (GPAT) serves as a crucial rate‐limiting enzyme, catalysing the conversion of glycerol‐3‐phosphate (G3P) and acyl‐CoA into lysophosphatidic acid (LPA) – the first committed step in triacylglycerol (TAG) synthesis (Takeuchi and Reue 2009). TAG is the main energy storage molecule in organisms. Its biosynthetic intermediates also function as membrane precursors and signalling molecules. Thus, the function of GPAT is indispensable for plant development. The subcellular localization, enzyme activity, and substrate specificity of GPATs determine their function (Wang 2004; Xu et al. 2023). Notably, most GPATs implicated in pollen development are localized to the endoplasmic reticulum (ER). Disruption of these ER‐localized GPATs in Arabidopsis (*AtGPAT1* and *AtGPAT6*), rice (*OsGPAT3*), and maize (*ZmMs33*/*ZmGPAT6*) leads to ER dysfunction and altered lipid composition, energy deficiency, and arrested anther development (Men et al. 2017; Xie et al. 2018; Zheng et al. 2003; Zhu, Li, et al. 2020; Zhu et al. 2019). Although the role of GPATs in pollen development is established, the functional coordination among GPAT family members under fluctuating temperatures remains unclear.

In this study, we identified a novel TGMS line, *Osgpat6.1*, which exhibits male sterility under HT and fertility under LT, along with brown glumes and a dwarf phenotype that enhances pollination efficiency. The *Osgpat6.1* showed impaired lipid metabolism, resulting in delayed tapetum degradation and defective pollen exine formation. By generating single, double, and triple mutants of *OsGPAT6.1*, *OsGPAT6.2*, and *OsGPAT6.3*, we demonstrated their redundant roles in regulating pollen development. Our results enhance the molecular understanding of TGMS and provide a valuable genetic resource for two‐line hybrid rice breeding.

## 2 Results

### 2.1 The *Osgpat6.1* Mutant Serves as a Stable TGMS Line

**FIGURE 1:**
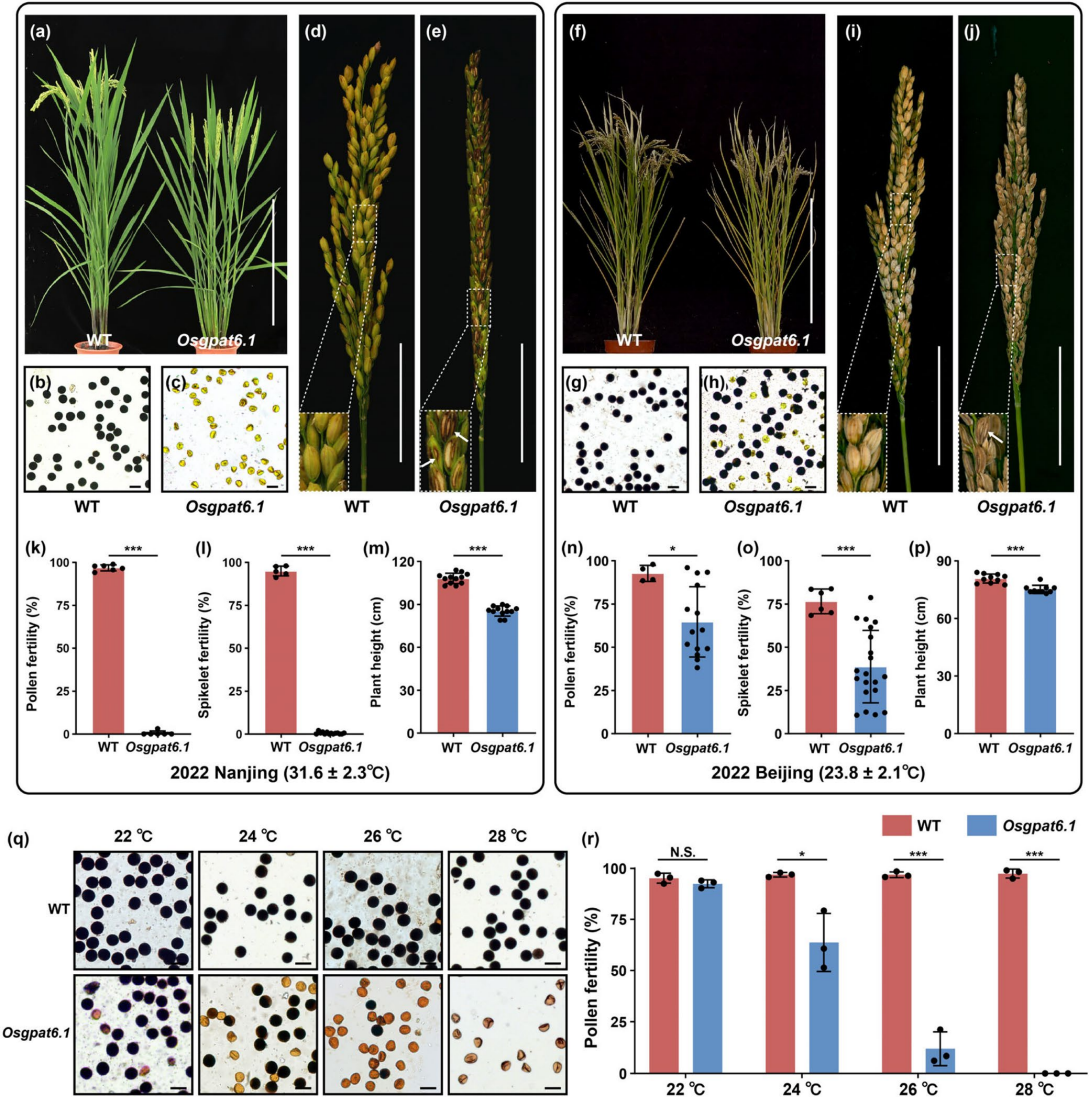
TGMS phenotype of *Osgpat6.1*. (a) Plant morphology of WT and *Osgpat6.1* under HT. Scale bar, 50 cm. (b, c) Pollen grains of WT (b) and *Osgpat6.1* (c) stained by I_2_‐KI solution under HT. Scale bar, 50 μm. (d, e) Panicle morphology of WT (d) and *Osgpat6.1* (e) under HT, the dashed box shows the enlarged spikelet and the arrow shows the brown glume in spikelet of *Osgpat6.1*. Scale bar, 5 cm. (f) Plant morphology of WT and *Osgpat6.1* under LT. Scale bar, 50 cm. (g, h) Pollen grains of WT (g) and *Osgpat6.1* (h) stained by I_2_‐KI solution under LT. Scale bar, 50 μm. (i, j) Panicle morphology of WT (i) and *Osgpat6.1* (j) under LT, the dashed box shows the enlarged spikelet and the arrow shows the brown glume in spikelet of *Osgpat6.1*. Scale bar, 5 cm. (k–m) Statistics of pollen fertility (k), spikelet fertility (l) and plant height (m) of WT and *Osgpat6.1* under HT. Values are means ± SD, *n* = 6 plants in (k), *n* = 5 (WT) and 14 (*Osgpat6.1*) plants in (l), *n* = 12 plants in (m). *p < 0.05, ***p < 0.001 by Student's *t*‐test. (n–p) Statistics of pollen fertility (n), spikelet fertility (o) and plant height (p) of WT and *Osgpat6.1* under HT. Values are means ± SD, *n* = 4 (WT) and 14 (*Osgpat6.1*) plants in (n), *n* = 6 (WT) and 20 (*Osgpat6.1*) plants in (o), *n* = 10 plants in (p). *p < 0.05, ***p < 0.001 by Student's *t*‐test. (q) Pollen grains of WT and *Osgpat6.1* stained by I_2_‐KI solution under different temperatures in the artificial climate chamber. Scale bar, 50 μm. (r) Statistics of pollen fertility of WT and *Osgpat6.1* under different temperatures in the artificial climate chamber. Scale bar, 50 μm. Shown as mean ± SD, *n* = 3. “N.S.” denotes no significant difference, *p < 0.05, ***p < 0.001 by Student's *t*‐test.

To identify novel environmentally sensitive male sterile mutant, *Osgpat6.1* was isolated from a rice EMS‐mutagenized ( *Oryza sativa* ssp. *japonica* L. cv. NJ 4) mutant library. Under HT conditions, *Osgpat6.1* exhibited pale yellow anthers and was completely male sterile, leading to spikelet abortion (Figure 1a–e,k,l; Figure S1a–f). In contrast, under LT, both pollen and spikelet fertility were partially restored to the wild type (WT) (Figure 1f–j,n,o). The *Osgpat6.1* mutant also displayed additional phenotypic traits, including reduced plant height (Figure 1m,p) and brown glumes (Figure 1e,j). These characteristics enhance its utility by improving pollination, reducing lodging, and enabling visual removal of off‐types in hybrid seed production.

To determine the critical sterility‐inducing temperature of *Osgpat6.1*, both WT and *Osgpat6.1* were subjected to different temperatures, and pollen fertility was measured. Pollen of *Osgpat6.1* was completely sterile at 28°C, and its fertility gradually increased as temperature dropped from 26°C to 22°C, with fully recovery observed at temperatures below 24°C (Figure 1q,r). These results indicate that *Osgpat6.1* was a stable TGMS line.

### 2.2 Delayed Tapetum PCD and Abnormal Pollen Wall Formation in *Osgpat6.1*

To characterize the anther abortion phenotype in *Osgpat6.1*, scanning electron microscopy (SEM) revealed that both WT and *Osgpat6.1* mature anthers exhibited characteristic ridge‐like ornamentations (Figure S1g–l), but *Osgpat6.1* pollen had slightly reduced in sporopollenin deposition compared with WT (Figure S1m–p). Eosin staining confirmed normal embryo sac development in *Osgpat6.1* (Figure S2a,b,e). Saturation pollination of WT pistils with *Osgpat6.1* pollen failed to produce seeds (Figure S2c,f). In contrast, pollination with WT pollen restored seed‐setting rates in *Osgpat6.1* (Figure S2d,f), conclusively demonstrating that the sterility phenotype results from male gametophytic defects.

**FIGURE 2:**
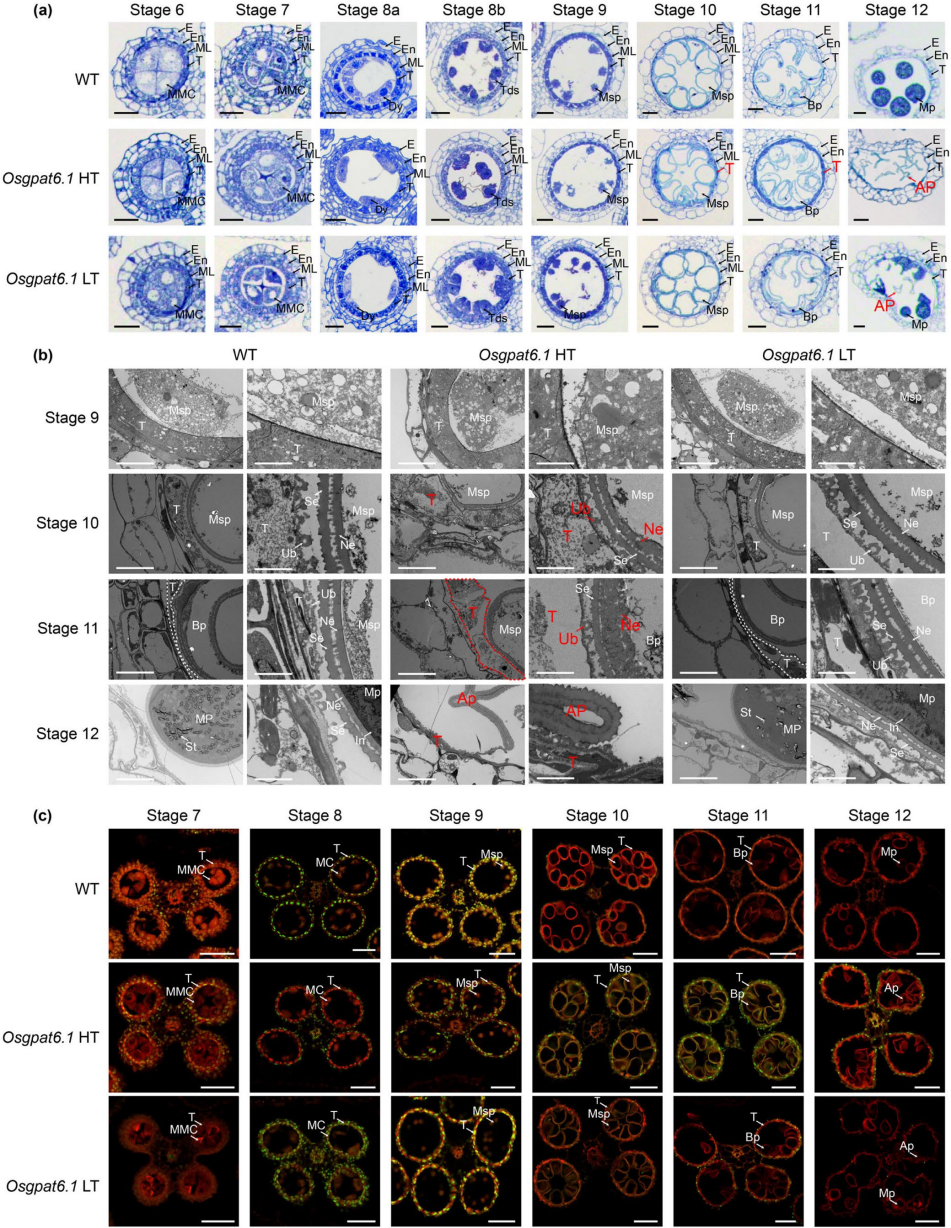
Delayed degradation in tapetum cells of *Osgpat6.1*. (a) Semi‐thin sections of anther locules at stage 6 to 12 of WT and *Osgpat6.1* under HT and LT. Scale bar, 20 μm. (b) Transmission electron micrographs of anthers and pollens at stage 9 to 12 of WT and *Osgpat6.1* under HT and LT. Scale bars, 10 μm in the first, third and fifth columns, and 2 μm in the second, fourth and sixth columns. (c) TUNEL analyses of anthers at stage 7 to 12 of WT and *Osgpat6.1* under HT and LT. Green fluorescence indicates TUNEL signal and red fluorescence indicates Propidium iodide staining. Scale bar, 50 μm. The red arrows or letters indicate the tapetum with delayed degradation or the abortive pollen in (a, b), while the dashed box highlights the tapetal cells (b). Stage 6, meiocyte mother cell stage; Stage 7, meiosis prophase I stage; Stage 8, meiosis stage; Stage 8a, dyad stage; Stage 8b, tetrad stage; Stage 9, early microspore stage; Stage 10, vacuolated microspore stage; Stage, bicellular pollen stage; Stage, tricellular pollen stage. Ap, abortive pollen; Bp, bicelullar pollen; Dy, dyad cell; E, epidermis; En, endothecium; MC, meiosis cell; Ml, middle layer; MMC, meiocyte mother cell; Mp, mature pollen; Msp, microspore; Ne, nexine; Se, sexine; St, starch granules; T, tapetum; Tds, tetrads; Ub, Ubisch body.

DAPI staining was performed to examine whether meiosis proceeds normally in *Osgpat6.1*. Similar to WT, *Osgpat6.1* exhibited normal meiotic chromosome behaviour (Figure S3), indicating that meiotic pollen mother cells (PMCs) were unaffected. Semi‐thin section analysis revealed no difference in anther structure between WT and *Osgpat6.1* from stages 6 to 9 under HT (Figure 2a). However, from stage 10, tapetum degradation proceeded rapidly in WT but was delayed in *Osgpat6.1*, with residual tapetum remnants persisting at stage 12 (Figure 2a). Under LT, tapetum degradation in *Osgpat6.1* was completed by stage 12, similar to WT (Figure 2a).

Transmission electron microscopy (TEM) demonstrated that at stage 9, the tapetum was condensed and underwent significant degradation in both WT and *Osgpat6.1* (Figure 2b). From stages 10 to 11, tapetum degradation proceeded normally in WT but was markedly delayed in *Osgpat6.1*, accompanied by irregular thickening of the nexine (Figure 2b). By stage 12, WT tapetum had fully degraded, with intact sexine/nexine layers and abundant starch granules in pollen, whereas *Osgpat6.1* retained residual tapetum remnants, resulting in pollen shrinkage and aberrant wall structure (Figure 2b). Similar to WT, tapetum degradation and structure of pollen wall were restored in *Osgpat6.1* under LT (Figure 2b).

Terminal deoxynucleotidyl transferase‐mediated dUTP nick‐end labeling (TUNEL) assay showed that in WT anthers, PCD‐associated fluorescence signals (green) appeared at stage 7. These signals peaked at stages 8–9, and then gradually diminished (Figure 2c). Under HT, *Osgpat6.1* exhibited a similar temporal onset of PCD signals (stage 7–9), but with significantly reduced intensity compared to WT, followed by persistent signals through stages 10 to 12 (Figure 2c). Under LT, PCD signals in *Osgpat6.1* were comparable to WT, which was consistent with the cytological observation. Collectively, these results suggest that delayed tapetum degradation under HT underlies male sterility in *Osgpat6.1* (Figure 2c).

### 2.3 Map‐Based Cloning of the *Osgpat6.1* Locus

**FIGURE 3:**
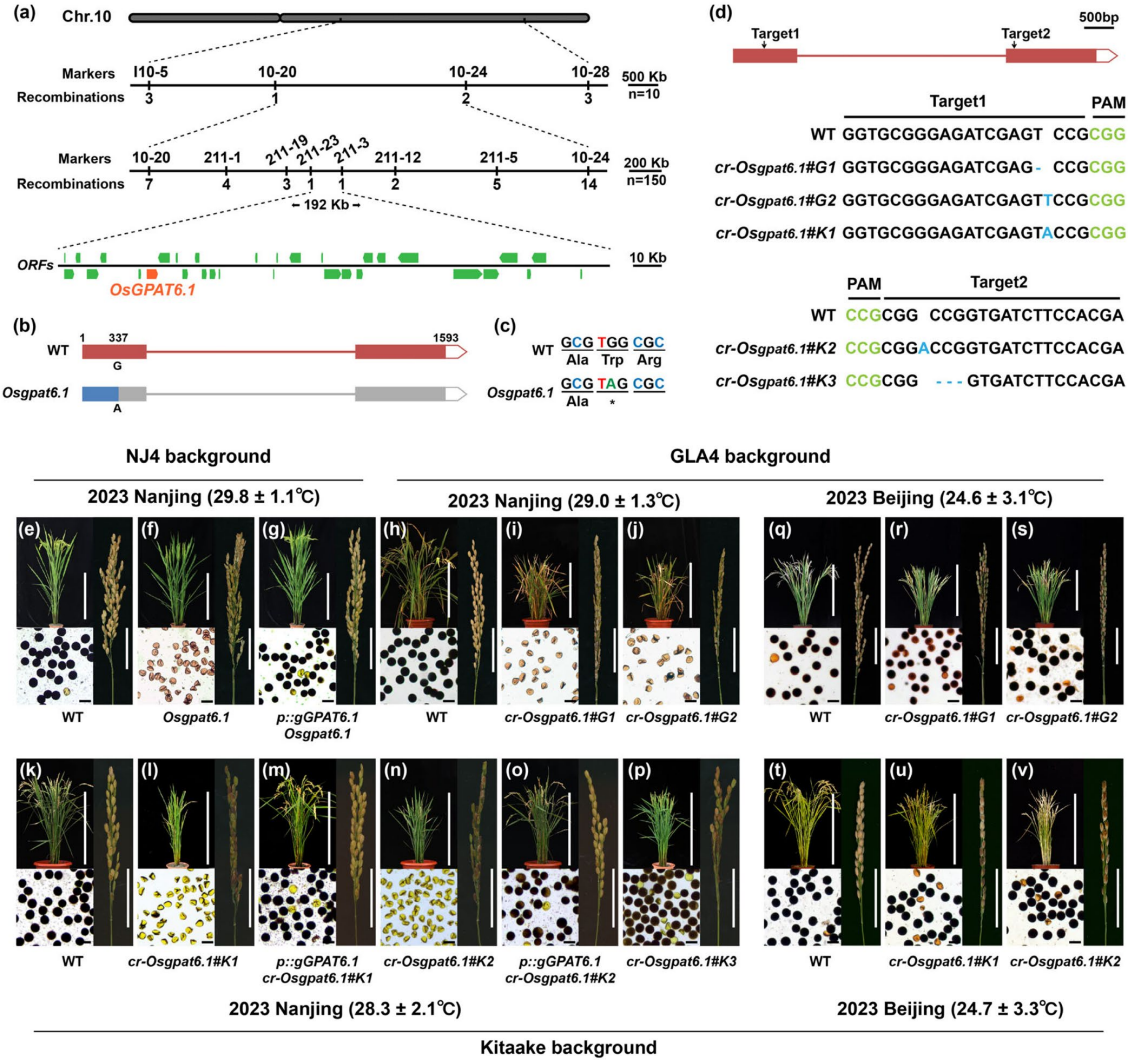
Cloning and identification of *Osgpat6.1*. (a) Fine mapping of the *Osgpat6.1* locus. The *Osgpat6.1* locus was initially mapped to a region on the long arm of chromosome 10 between molecular markers 10‐20 and 10‐24 using 10 sterile plants. Whereafter, fine mapping of *Osgpat6.1* was performed within a 192‐kb interval between markers 211‐23 and 211‐3 using 150 plants. 31 *ORFs* were predicted within this interval. The numbers beneath the vertical lines and blocks at the bottom represent the recombinant plants and candidate genes, respectively. Chr, chromosome; *ORFs*, *open reading frames*. (b, c) Genomic structure and the mutation site of *OsGPAT6.1*. Exons are shown as boxes, and introns as lines. The *Osgpat6.1* has a G to A single nucleotide polymorphism change (b), which led to translation terminated prematurely (c). (d) Schematic representation of CRISPR/Cas9 targets sites. Target 1 is located in the first exon; *cr‐Osgpat6.1#G1* has a T base deletion, *cr‐Osgpat6.1#G2* has a T base insertion, and *cr‐Osgpat6.1#K1* has an A base insertion. Target 2 is located in the second exon, where *cr‐Osgpat6.1#K2* has an A base insertion, and *cr‐Osgpat6.1#K3* has a 3‐base deletion. (e–v) Gene function validation of *OsGPAT6.1*. (e–g) *Osgpat6.1* was rescued by the *OsGPAT6.1* genome sequence driven by the *OsGPAT6.1* native promoter under HT. Two independent knockout lines in the GLA4 background were male sterile under HT (h–j) and partial restoration of fertility under LT (q–s). Two independent knockout lines in the kitaake background were male sterile under HTs (l, n) and partial restoration of fertility under LT (t–v). And the *OsGPAT6.1* genome sequence driven by the *OsGPAT6.1* native promoter rescued those two lines under HT (m, o). Another 3‐bp deleted line showed a slight decrease in male fertility under HTs (p). Scale bars, 50 cm for the plant shot in the upper left corner and 50 μm for the pollen shot in the lower left corner and 5 cm for the panicle shot on the right side for (e–v) images.

Genetic analysis of an F_2_ population, derived from F_1_ plants (*OsGPAT6.1*/*Osgpat6.1*) exhibited a segregation ratio of 3:1 (fertile to sterile) with 178 fertile plants and 50 sterile plants (*χ* ^2^ = 1.146 < *χ* ^2^_0.05,1_ = 3.84) (Table S1). To map the *Osgpat6.1* locus, an F_2_ population was generated by crossing male sterile plants (*Osgpat6.1*) with *Oryza sativa* ssp. *aus* L. N22. Preliminary mapping placed the *Osgpat6.1* locus between InDel markers 10–20 and 10–24 on chromosome 10. Further fine‐mapping using male sterile plants from the F_2_ mapping population delimited the locus to a 192‐kb region between InDel markers 211–23 and 211–3. This region contains 31 predicted open reading frames (ORFs) (Figure 3a).

Based on bulked segregant analysis (BSA) of fertile and sterile F_2_ pools of the mapped 192‐kb interval, *ORF7* (*LOC_Os10g27330*) was identified as the only gene carrying a coding‐region SNP (Table S2). This SNP is a G‐to‐A transition in the first exon that causes premature translational termination (Figure 3b,c). *ORF7* encodes a glycerol‐3‐phosphate acyltransferase (GPAT) involved in lipid biosynthesis, which we designated as *OsGPAT6.1*. Genetic complementation using the construct *p:gGPAT6.1* (containing its native promoter and complete genome sequence) fully restored pollen fertility in *Osgpat6.1* under HT conditions (Figure 3e–g; Figure S4). Additionally, CRISPR/Cas9‐mediated knockout lines of *OsGPAT6.1* were generated in the Kitaake and GLA4 genetic backgrounds (Figure 3d). All knockout lines exhibited TGMS, showing sterility under HT but normal fertility under LT, along with dwarf stature and brown glumes, consistent with the *Osgpat6.1* mutant phenotype (Figure 3h–v; Figure S4). Furthermore, the *p:gGPAT6.1* construct also rescued pollen fertility in the *Osgpat6.1* mutant in the Kitaake background under HT (Figure 3m,o; Figure S4). All these results conclusively demonstrate that the mutation in *OsGPAT6.1* is the causal factor underlying the TGMS phenotype.

### 2.4 *OsGPAT6.1* Is Highly Expressed in the Tapetum and Encodes an ER‐Localized GPAT Enzyme

**FIGURE 4:**
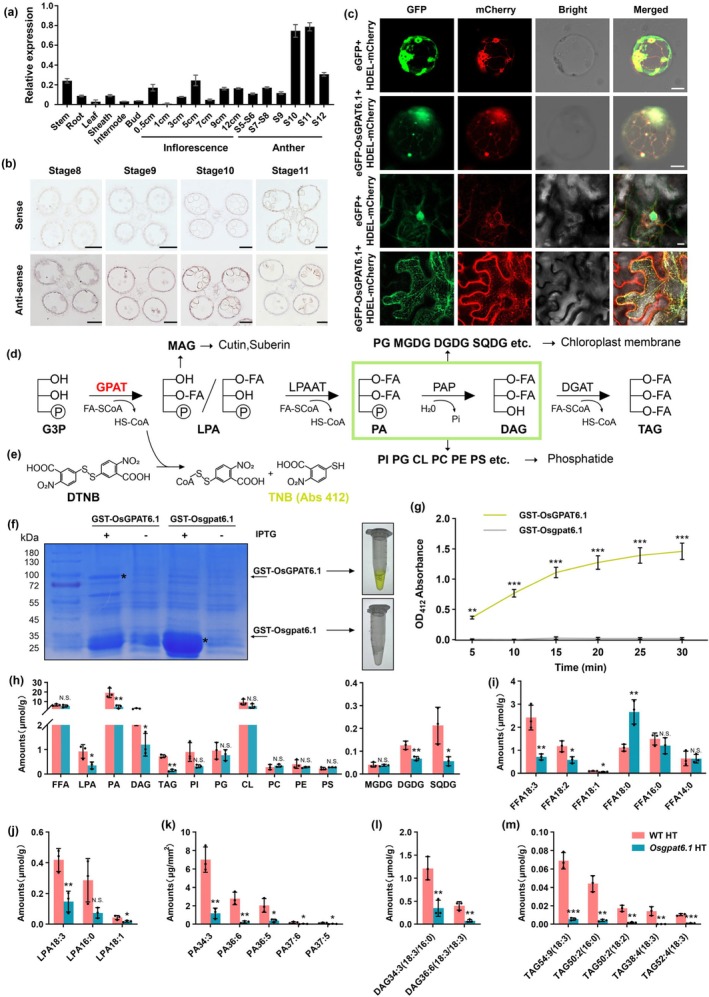
Expression pattern of *OsGPAT6.1* and molecular functions of OsGPAT6.1. (a) qRT‐PCR analysis of *OsGPAT6.1* in various tissues and developing stage of panicle of WT. Values are means ± SEM, *n* = 3. (b) In situ hybridization of *OsGPAT6.1* transcripts in different stages anthers of WT. (c) Subcellular localization assay of OsGPAT6.1 in rice protoplasts and tobacco leaf cells. Scale bar, 10 μm. HDEL‐mCherry was used as an ER marker. (d) Schematic representation of the of TAG biosynthesis, where GPAT is the enzyme for the first step of the reaction. The substrate FA‐SCoA and G3P catalysed by GPAT will produce LPA and release HS‐CoA. (e) Detection of the product of the above reaction can be used as an indicator of GPAT enzyme activity, the DTNB will react with HS‐CoA to produce yellow TNB. (f) Coomassie blue staining of SDS‐PAGE gel showing purified GST‐OsGPAT6.1 and GST‐Osgpat6.1 expressed in vitro followed by the addition of the GPAT substrate system. DTNB was added at the end of the reaction, which generated yellow TNB. (g) The Abs_412_ of the final product was measured as the concentration of TNB and was used to represent GPAT activity. Values are means ± SEM, *n* = 6. **p < 0.01, ***p < 0.001 by Student's *t*‐test. (h) Analysis of WT and *Osgpat6.1* anther during stage 12 of anther development total lipid component under HT. Analysis of WT and *Osgpat6.1* anther all FFA (i) and partial the most abundant LPA (j), PA (k), DAG (l) and TAG (m) component under HT. Values are means ± SD, *n* = 3. “N.S.” denotes no significant difference, *p < 0.05, ***p < 0.001 by Student's *t*‐test.

To investigate the expression profile of *OsGPAT6.1*, qRT‐PCR analysis showed low expression at stages 6–9 and peak expression at stages 10–11, coinciding with tapetum degradation (Figure 4a). In situ hybridization further revealed strong tapetum‐specific signals around stages 10 (Figure 4b). Subcellular localization assay using GFP‐tagged OsGPAT6.1 in rice protoplasts and *Nicotiana benthamiana* epidermal cells showed co‐localization with the ER marker HDEL‐mCherry, supporting ER localization (Figure 4c). Together, these results indicate that OsGPAT6.1 is an ER‐localized protein highly expressed in the tapetum and is likely involved in tapetum function.

Previous studies have demonstrated that GPAT enzymes catalyse the acylation of glycerol‐3‐phosphate (G3P) with fatty acyl‐CoA (FA‐CoA) to produce lysophosphatidic acid (LPA) and CoA‐SH, a key step in lipid biosynthesis (Figure 4d) (Ohlrogge and Browse 1995). GPAT activity of OsGPAT6.1 was assayed using a DTNB (5,5′‐dithiobis‐2‐nitrobenzoic acid)‐based method, in which HS‐CoA released from the acyltransferase reaction reacted with DTNB to produce the yellow compound 5‐thio‐2‐nitrobenzoic acid (TNB), detectable at 412 nm (Figure 4e). Recombinant GST‐tagged proteins were generated by fusing the coding sequences of *OsGPAT6.1* and a truncated version corresponding to the *Osgpat6.1* mutant. These constructs were expressed in *Escherichia coli* (DE3 strain) and subsequently purified (Figure 4f). In vitro enzyme assays revealed that GST‐OsGPAT6.1 rapidly produced the yellow TNB product, with absorbance at 412 nm increasing over time, whereas GST‐Osgpat6.1 showed no detectable activity (Figure 4f,g). These results confirm that the mutation in *Osgpat6.1* abolishes GPAT enzymatic function.

### 2.5 OsGPAT6.1 Is Crucial for Lipid Metabolism in Rice Anthers

LPA, the product of GPAT activity, serves as a key intermediate in triacylglycerol (TAG) biosynthesis. Other intermediates in this pathway, including phosphatidic acid (PA) and diacylglycerol (DAG), serve as important signalling molecules, and their metabolic derivatives are critical components of cellular membranes (Figure 4d) (Hong et al. 2016; Maraschin et al. 2019; Ohlrogge and Browse 1995; Wang et al. 2006; Zhang et al. 2004). To investigate the metabolic consequences of *Osgpat6.1*, lipidomic analysis of mature anthers from both WT and *Osgpat6.1* was performed using gas chromatography–mass spectrometry.

The *Osgpat6.1* mutant showed significant reductions in total content and major molecular species of LPA, PA, DAG, and TAG under HT (Figure 4h–m). These reductions indicate a global impairment of glycerolipid biosynthesis in *Osgpat6.1* anthers under HT. Although the total free fatty acids (FFAs)—substrates for GPAT—were not significantly different between WT and *Osgpat6.1*, the fatty acid composition was altered in *Osgpat6.1*. Specifically, *Osgpat6.1* showed decreased α‐linolenic acid (C18:3), linoleic acid (C18:2), and oleic acid (C18:1), with increased stearic acid (C18:0) (Figure 4h,i). These changes suggest that *OsGPAT6.1* preferentially utilizes saturated C18 fatty acids during acyltransferase activity. Furthermore, quantitative analysis revealed significant reductions in key chloroplast membrane lipids, particularly digalactosyldiacylglycerol (DGDG) and sulfoquinovosyldiacylglycerol (SQDG) in *Osgpat6.1* under HT (Figure 4h). These changes may compromise chloroplast membrane integrity and contribute to impaired pollen development. Although the total amounts of other lipid classes such as phosphatidylinositol (PI), phosphatidylglycerol (PG), cardiolipin (CL), phosphatidylcholine (PC), phosphatidylethanolamine (PE), phosphatidylserine (PS), and monogalactosyldiacylglycerol (MGDG) remained largely unchanged, their molecular compositions were significantly altered under HT (Figure 4h; Table S4).

Compared to HT, the lipid profile of *Ospgat6.1* anthers under LT shows a marked improvement, with significant increases in all direct products of the TAG pathway, including LPA, PA, DAG, and TAG, which almost reach the levels observed in the WT (Figure S5; Table S5). Additionally, both DGDG and SQDG show significant increases in *Ospgat6.1* under LT. These changes suggest that LT conditions help to restore lipid metabolism, particularly in the TAG biosynthesis pathway, which may contribute to the recovery of fertility in *Ospgat6.1* under low‐temperature conditions.

To further explore whether metabolism‐related genes are affected, we performed RNA‐seq analysis and identified 1501 up‐regulated and 1480 down‐regulated genes in *Osgpat6.1* (Figure S6a). KEGG enrichment analysis revealed enrichment in metabolic and biosynthesis of secondary metabolites, indicating extensive metabolic reprogramming in *Osgpat6.1* (Figure S6b). Several lipid metabolism pathways, including phenylpropanoid biosynthesis, fatty acid elongation, and cutin, suberine, and wax biosynthesis that are known to be involved in pollen wall formation, were significantly enriched (Figure S6b) (Ariizumi and Toriyama 2011; Chen et al. 2024; Fellenberg and Vogt 2015). These results are consistent with the disrupted anther development and impaired pollen wall integrity in *Osgpat6.1*. Together, these findings indicate that comprehensive lipid metabolic disturbances, stemming from impaired GPAT activity in *Osgpat6.1*, underlie the defective pollen development.

### 2.6 Redundant Roles of *OsGPAT6.1*, *OsGPAT6.2*, and *OsGPAT6.*3 in Regulating Pollen Development

**FIGURE 5:**
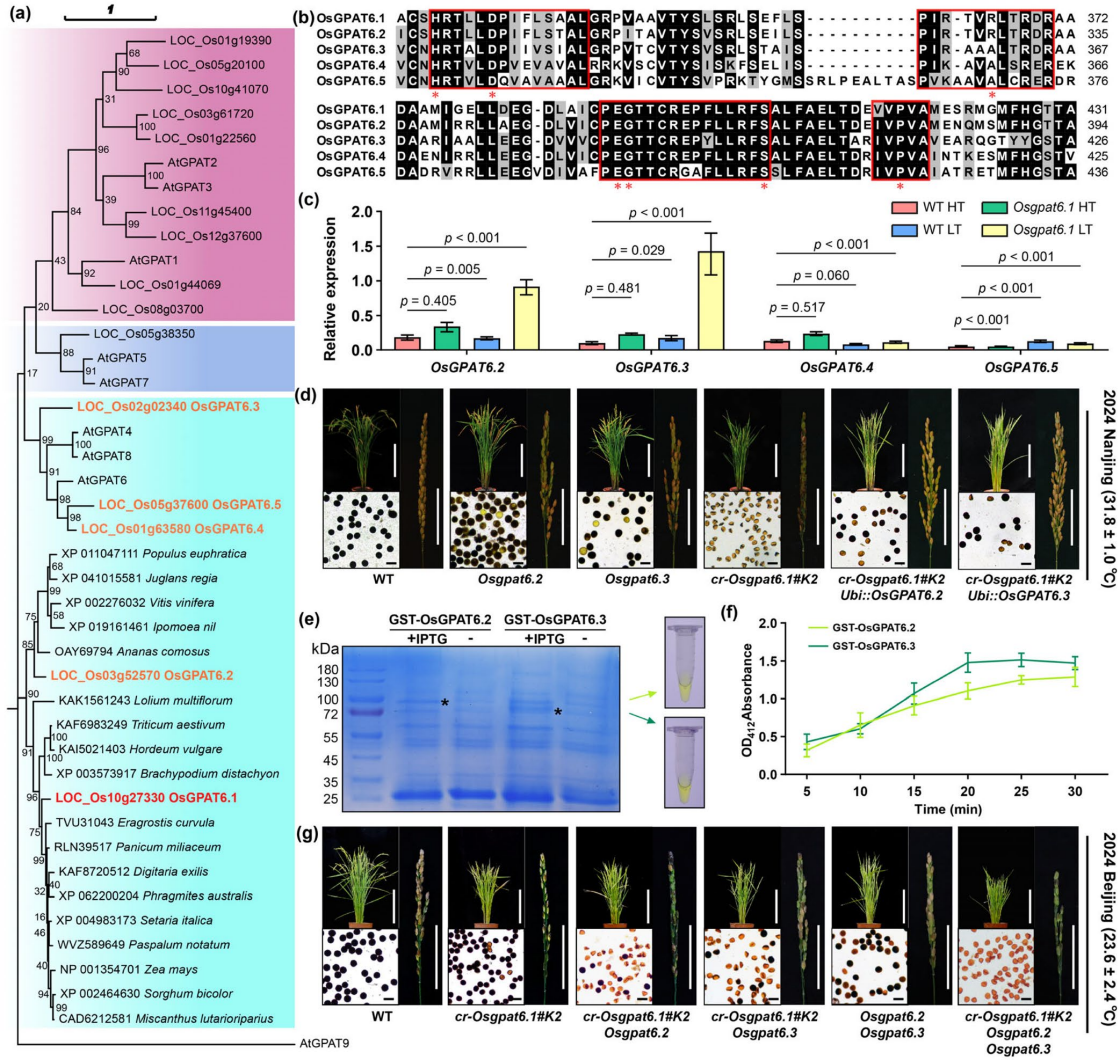
*OsGPAT6.1* functionally redundantly regulates rice pollen development with *OsGPAT6.2* and *OsGPAT6.3*. (a) Phylogenetic analysis between GPATs in rice and Arabidopsis and OsGPAT6.1 homologues in 18 other species. OsGPAT6.1 belongs to the subfamily of GPAT4/6/8 in Arabidopsis. (b) Amino acid sequence of the conserved acyltransferase domain (AT) of OsGPAT6.1 with its four homologues. Asterisks denote the amino acids essential for GPAT enzyme activity. (c) qRT‐PCR analysis of four *OsGPAT6.1* homologous genes under various conditions. Values are means ± SEM, *n* = 3. (d) WT and *cr‐Osgpat6.1#K2* were used as controls, phenotypes of *Osgpat6.2* and *Osgpat6.3* knockout lines as well as *Osgpat6.1*#K2 *Ubi::OsGPAT6.2* and *Osgpat6.1*#K2 *Ubi::OsGPAT6.3* lines in the kitaake background under HT. (e) Coomassie blue staining of SDS‐PAGE gel showing purified GST‐OsGPAT6.2 and GST‐Osgpat6.3 expressed in vitro followed by the addition of the GPAT substrate system and DTNB, indicating that both OsGPAT6.2 and OsGPAT6.3 have GPAT enzymatic activity. (f) The Abs_412_ of the final product was measured as the concentration of TNB and was used to represent GPAT activity. Values are means ± SEM, *n* = 4. (g) WT and *cr‐Osgpat6.1#K2* were used as controls, phenotypic of three double mutants *cr‐Osgpat6.1#K2Osgpat6.2*, *cr‐Osgpat6.1#K2Osgpat6.3*, *Osgpat6.2Osgpat6.3* and one triple mutant *cr‐Osgpat6.1#K2Osgpat6.2Osgpat6.3* in the kitaake background under LT.

Phylogenetic analysis revealed that OsGPAT6.1 belongs to the Arabidopsis GPAT4/6/8 subfamily. Four rice homologues within the same clade were identified and designated as OsGPAT6.2 to OsGPAT6.5 (Figure 5a). All five proteins contain a highly conserved acyltransferase (AT) domain, suggesting potential functional redundancy (Figure 5b). Expression analysis showed that *OsGPAT6.2* and *OsGPAT6.3* were significantly upregulated in the anthers of *Osgpat6.1* mutant under LT conditions, indicating that their induction is specifically triggered by LT in *Osgpat6.1* mutant background. In contrast, *OsGPAT6.4* and *OsGPAT6.5* exhibited consistently low expression levels (Figure 5c). To assess the roles of these GPATs, especially *OsGPAT6.2* and *OsGPAT6.3* in TGMS regulation, CRISPR/Cas9‐mediated knockout lines were generated (Figure S7). Under HT, *Osgpat6.2* and *Osgpat6.3* mutants showed reduced pollen fertility and spikelet fertility (Figure 5d; Figure S8). These results demonstrate that *OsGPAT6.2* and *OsGPAT6.3* are required for pollen development. Subsequent in vitro enzyme assays, using the same DTNB‐based method described for OsGPAT6.1, confirmed that both OsGPAT6.2 and OsGPAT6.3 possess GPAT enzymatic activity (Figure 5e,f). Structural predictions via AlphaFold3 revealed that OsGPAT6.2 and OsGPAT6.3 share highly similar three‐dimensional architectures with OsGPAT6.1, showing strong conservation in the key transmembrane and acyltransferase domains (Figure S9) (Abramson et al. 2024). Subcellular localization in *N. benthamiana* epidermal cells revealed that both OsGPAT6.2 and OsGPAT6.3 were localized on ER, consistent with OsGPAT6.1 (Figure S10). In situ hybridization further revealed that both *OsGPAT6.2* and *OsGPAT6.3* were expressed in the tapetum from stage 8 to 11, overlapping with the expression window of *OsGPAT6.1* (Figure S11).

Based on the above findings, we propose that *OsGPAT6.1*, *OsGPAT6.2*, and *OsGPAT6.3* function redundantly in pollen development. To test this, overexpression of *OsGPAT6.2* or *OsGPAT6.3* driven by the *Ubi* promoter were generated in the *Osgpat6.1* mutant background. Under HT, the *cr‐Osgpat6.1#K2 Ubi::OsGPAT6.2* and *cr‐Osgpat6.1#K2 Ubi::OsGPAT6.2* both partially restored fertility and plant height (Figure 5d; Figure S8). multi‐gene CRISPR/Cas9‐mediated knockout lines targeting different combinations of these genes were generated in Kitaake background (Figure S7). Under LT, the *cr‐Osgpat6.1#K2Osgpat6.2* and *cr‐Osgpat6.1#K2Osgpat6.3* mutants failed to restore pollen fertility compared to *cr‐Osgpat6.1#K2*, and *cr‐Osgpat6.1#K2Osgpat6.2Osgpat6.3* exhibited completely male sterile (Figure 5g; Figure S8). In addition, similar results were observed for double and triple mutants in NJ4 background (Figure S12). both double mutants *Osgpat6.1Osgpat6.2* and *Osgpat6.1Osgpat6.3* already displayed complete male sterility while the triple mutant *Osgpat6.1Osgpat6.2Osgpat6.3* showed even no pollen grains (Figure S12). Collectively, these data demonstrate that *OsGPAT6.1*, *OsGPAT6.2*, and *OsGPAT6.3* collectively provide the GPAT enzymatic activity necessary for pollen development, and that this redundant function is essential for the manifestation of the TGMS phenotype.

### 2.7 Potential Application of *Osgpat6.1* in the Two‐Line Hybrid Breeding System

Given its TGMS phenotype, the potential utility of *Osgpat6.1* for two‐line hybrid breeding system was evaluated. The mutant was crossed with five cultivars (T65, Dular, N22, DJY, and NIP), and the field performance of the resulting hybrids and their parental lines was assessed. Hybrids harbouring *Osgpat6.1* (e.g., *Osgpat6.1* × T65, etc.) exhibited comparable heterosis to corresponding WT crosses (NJ4 × T65, etc.), with increased plant height, tiller number, and grain number per panicle relative to parents. Moreover, no significant differences in key agronomic traits were detected between hybrids carrying *Osgpat6.1* and their WT counterparts (Figure S13). These results suggest that hybrids carrying heterozygous *Osgpat6.1* do not affect hybrid vigour, confirming the suitability of *Osgpat6.1* has potential for application in the two‐line hybrid breeding system. Beyond its TGMS phenotype, *Osgpat6.1* possesses agronomically favourable traits that facilitate breeding. The semi‐dwarf stature of *Osgpat6.1* not only enhances lodging resistance but also provides an optimal plant height for efficient pollen reception during outcrossing, a critical feature for maternal sterile lines. Additionally, the distinctive brown glume coloration of *Osgpat6.1* serves as a convenient phenotypic marker, partially reducing the dependency on molecular markers for selection during breeding. Genetic analyses established *OsGPAT6.1* as the primary gene controlling TGMS, with *OsGPAT6.2* and *OsGPAT6.3* acting redundantly to fine‐tune pollen development (Figure 5g; Figure S12).

Considering the variation in photo‐thermal conditions across rice‐growing regions and differences in genetic backgrounds, targeted manipulation (knockout, knockdown, or overexpression) of *OsGPAT6.2* and *OsGPAT6.3* can compensate for the instability of the TGMS phenotype when relying solely on the *OsGPAT6.1* mutant (*Osgpat6.1*). It provides a strategy to fine‐tune the TGMS phenotype for local environmental adaptability and genetic compatibility.

## 3 Discussion

**FIGURE 6:**
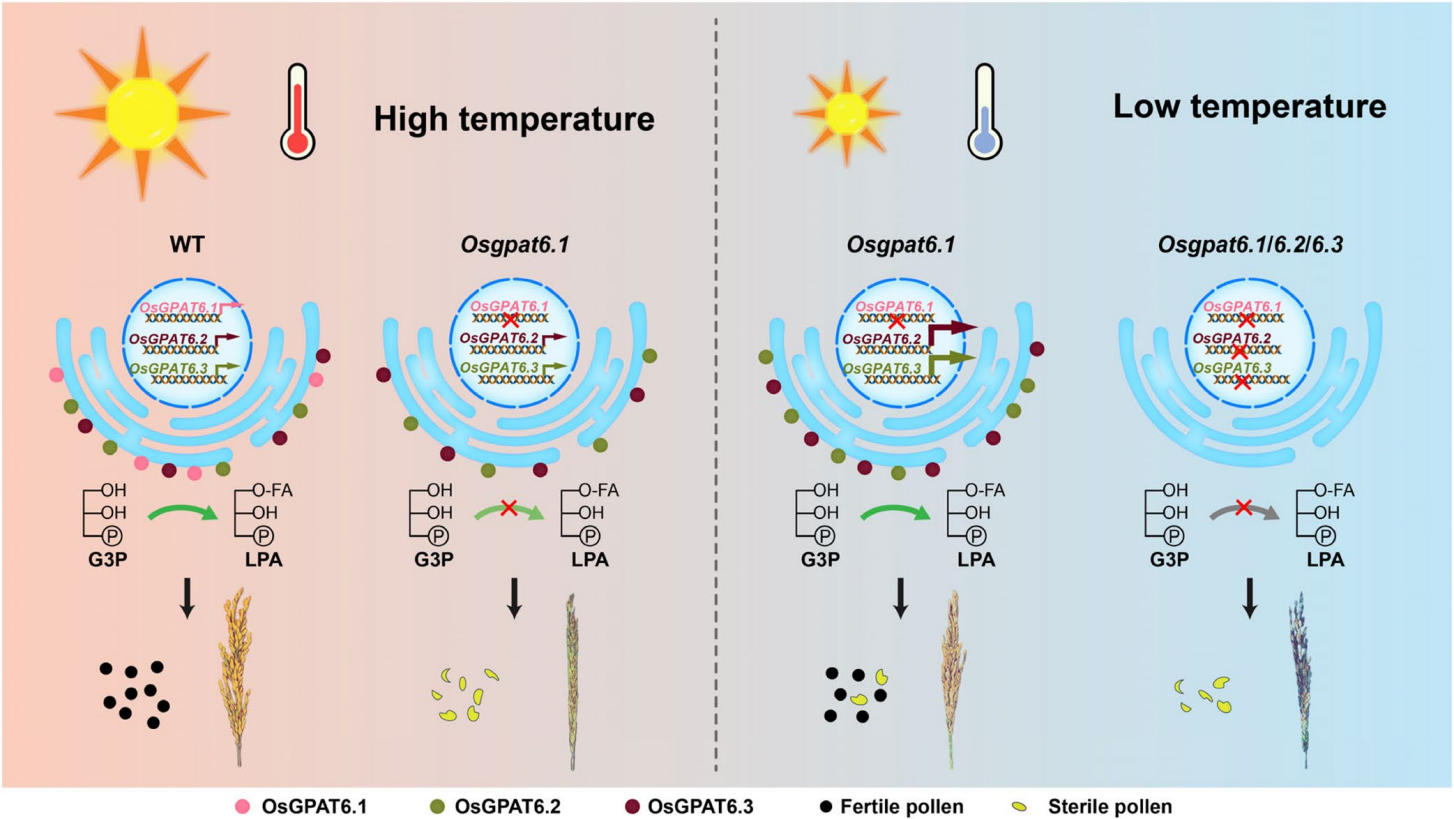
A proposed model of TGMS in rice through functional compensation by glycerol‐3‐phosphate acyltransferase OsGPAT6.1, OsGPAT6.2, and OsGPAT6.3. Under HT, OsGPAT6.1 functions together with OsGPAT6.2 and OsGPAT6.3 to fulfil GPAT activity and maintain the stability of lipid metabolism, resulting in fertile pollen. In the *Osgpat6.1* mutant, OsGPAT6.2 and OsGPAT6.3 alone are insufficient to maintain the stability of lipid metabolism, leading to sterile pollen. Under LT, the expression levels of *OsGPAT6.2* and *OsGPAT6.3* are increased in *Osgpat6.1* mutants; OsGPAT6.2 and OsGPAT6.3 are sufficient to maintain the stability of lipid metabolism, resulting in partially fertile pollen. In the *Osgpat6.1Osgpat6.2Osgpat6.3* triple mutant, GPAT activity was completely lost, resulting in sterile pollen.

In this study, we report the molecular cloning and functional dissection of *Osgpat6.1*, which represents a novel TGMS line exhibiting complete male sterility under HT, while partially restoring fertility under LT. In addition to the stable TGMS phenotype, *Osgpat6.1* displays brown glumes and dwarf stature, traits that may beneficial for hybrid rice breeding. Genetic analysis revealed that the causal gene, *OsGPAT6.1*, encodes a GPAT localized to the ER and plays a critical role in lipid metabolism during development of anther. Disruption of *OsGPAT6.1* alters lipid composition, delays PCD of the tapetum, and impairs pollen exine formation, resulting in male sterility. Partial restoration of fertility under LT is likely due to functional redundancy with its homologues *OsGPAT6.2* and *OsGPAT6*.3 (Figure 6). These features highlight the potential of *Osgpat6.1* as a promising resource for two‐line hybrid rice breeding.

In eukaryotes, ER‐localized GPATs are crucial for the synthesis of LPA, PA, and TAG, which serve as precursors in the biosynthesis of phospholipid, glycolipid, and triglyceride. These lipids are essential for membrane lipid metabolism, signal transduction, and cytoskeleton remodelling (Hong et al. 2016; Ohba et al. 2013; Takeuchi and Reue 2009; Wang et al. 2006; Zhang et al. 2004). Moreover, GPAT‐mediated lipid metabolism is indispensable for ER integrity. For example, dysfunction of *AtGPAT1* and *AtGPAT6* alters ER morphology in Arabidopsis (Li et al. 2012; Zheng et al. 2003). Our results indicate that ER‐localized OsGPAT6.1 possesses GPAT activity, catalysing the biosynthesis of LPA from G3P. The content of LPA, PA, and TAG was significantly reduced in *Osgpat6.1* anthers. We speculated that this disruption affects signal transduction and lipid synthesis pathways related to tapetum development. This leads to altered ER morphology, impaired protein synthesis and transport, and ultimately delayed tapetum degradation. Consistent with our findings, mutants of *AtGPAT1*, *AtGPAT6*, *OsGPAT3*, and *ZmMs33* also showed abnormal degradation of anther tapetum, indicating that GPAT‐mediated lipid metabolism represents a conserved regulatory mechanism governing tapetum development across plant species (Li‐Beisson et al. 2009; Li et al. 2012; Men et al. 2017; Sun et al. 2018; Zhang et al. 2018; Zheng et al. 2003; Zhu, Li, et al. 2020).

Functional redundancy among genes is widespread in nature and plays a vital role in enhancing genetic stability and environmental adaptability (Fay and Han 2000; Gottlieb 2003; Louca et al. 2018; Peng 2019; Sambamoorthy and Raman 2018; Sanfaçon 2015; Schilling et al. 2019; Soza et al. 2016; Veley and Michaels 2008). Previous studies have revealed that LT slows the development of anther, thereby regulating the temperature and light‐sensitive phenotype of pollen (Zhang et al. 2020; Zhu, Lou, et al. 2020). LT not only extends the time window for material accumulation by delaying pollen development but also provides a critical opportunity for functional compensation in defective mutants. Such compensatory mechanisms may arise from residual activity of the mutant protein (Han et al. 2023; Wu et al. 2022; Zhang et al. 2022) or through functional redundancy of homologous genes. Our study demonstrates that the fertility restoration of the *Osgpat6.1* mutant under LT entirely depends on functional compensation by its homologous genes *OsGPAT6.2* and *OsGPAT6.3*. When these homologous genes are simultaneously inactivated, even LT fails to rescue the fertility defects of the mutant (Figure 6). This is consistent with previous results showing *TMS10*, which encodes a leucine‐rich repeat receptor‐like kinase (LRR‐RLK), and its homologue *TMS10L* redundantly regulating temperature‐sensitive pollen fertility in rice. However, unlike *OsGPAT6.1* homologues, *TMS10L* single mutants do not exhibit reduced fertility. Our discovery of three GPAT genes underscores the critical role of functional redundancy in restoring TGMS fertility under LT. Redundant gene function, combined with LT‐induced slowing of development of anther, likely regulates TGMS by maintaining lipid metabolism essential for tapetum function and pollen wall formation. This redundancy network buffers defective gene function, stabilizing fertility transitions. Leveraging these homologous genes offers a promising strategy for precise TGMS regulation, enabling the development of environmentally adaptable and genetically compatible two‐line hybrid rice varieties.

Compared to the three‐line breeding system, the two‐line breeding system offers several advantages, including a simplified breeding process, improved breeding efficiency, and broader applicability of sterile resources. However, it also faces challenges such as the environmental sensitivity of sterile lines. In this context, the most important advantage of the *Osgpat6.1* mutant is its stable TGMS, ensuring reliable sterility during hybrid seed production under HT. In addition, traits such as reduced plant height and brown glumes enhance pollination efficiency, reduce lodging risk, and allow visual identification and removal of off‐type plants—advantages not present in the widely used *tms5* sterile lines (Zhou et al. 2014). Furthermore, introducing the *Osgpat6.1* allele into other rice varieties consistently produces stable TGMS, and dysfunction of *Osgpat6.1* does not affect the phenotype of the hybrid plants. Together, these findings underscore the strong breeding potential of *Osgpat6.1* and provide a foundation for large‐scale field evaluations to further explore its utility in two‐line hybrid rice breeding.

## 4 Materials and Method

### 4.1 Plant Materials

The *Osgpat6.1* mutant used in this study was obtained from an EMS‐mutagenized population of the *japonica* cv. NJ4. Knockout plants of *Osgpat6.1* and its homologous genes were generated in the *Oryza sativa subsp. japonica* cv. Kitaake or Guangluai 4 background. A series of multiple gene knockout mutants were generated in the *Oryza sativa subsp. japonica* cv. Kitaake background. All rice plants were grown in paddy fields at Nanjing Agricultural University (Nanjing, China) and Chinese Academy of Agricultural Sciences (Beijing, China) for further phenotypic characterization.

### 4.2 Temperature Treatment for Rice

WT and *Osgpat6.1* mutant (NJ4 background) rice were grown in the field (Chinese Academy of Agricultural Sciences, Beijing, China) or in greenhouse settings until the premeiotic stage, and then transferred to growth chambers maintained at average daytime temperatures of 22°C, 24°C, 26°C, or 28°C and 70% relative humidity until flowering. Rice anthers were collected at appropriate developmental stages and stained with 1% KI‐*I*_2_ solution for further analysis.

### 4.3 Plasmid Construction

To generate knock out mutants of *Osgpat6.1* and its homologous genes, 20‐bp target sequences were synthesized and cloned into the pOs‐Cas9 vector (Miao et al. 2013). The CRISPR/Cas9 constructs were introduced into *O. japonica* cv. Kitaake and *O. indica* cv. Guangluai 4 via Agrobacterium‐mediated transformation. To investigate the role of *Osgpat6.1* and its homologous genes in regulation of TGMS, additional vectors targeting multiple genes were constructed and introduced into Kitaake. The primer sequences used in this study are provided in Table S3.

### 4.4 Semi‐Thin Sections

Young panicles were collected and fixed in Carnoy's solution (ethanol: acetic acid = 3: 1) for at least 24 h at room temperature. The young panicles were rinsed twice with PBS buffer, each time for 20 min, and then dehydrated in a series of ethanol concentrations (70%, 80%, 90%, and 95%). The ethanol solution was removed, and the panicles were transferred to a staining solution (ethanol: acetone = 1: 1, which contained eosin) and acetone for at least 1 h, respectively. The young panicles were then treated with a gradient solution of resin and acetone (acetone: resin = 2:1, acetone: resin = 1:1, acetone: resin = 1: 2, and resin, with each step lasting at least 3 h) and then embedded in a mould. After trimming and sectioning to poly‐L‐lysine‐coated slides, the sections were stained with 0.5% toluidine blue staining solution and observed using a Zeiss Fluorescence Microscope.

### 4.5 TUNEL Analyses

TUNEL analyses were performed as previously described (Bai et al. 2019). To examine tapetum degradation in WT and the *Osgpat6.1* mutant, TUNEL assays were performed using a TUNEL Kit (Promega). Young panicles of different developmental stages were collected and fixed in FAA solution (100 mL of FAA solution consists of 5 mL of formalin (38% formaldehyde), 5 mL of acetic acid, and 90 mL of 70% alcohol). Paraffin sections of the anthers were prepared, and the subsequent experimental procedures were conducted according to the TUNEL Kit instructions. The green fluorescence and red fluorescence were examined using a Zeiss LSM980 laser scanning microscope with a × 10 objective (0.4 numerical aperture). Images were captured using Zen 3.3 software.

### 4.6 Phylogenetic Analysis

Sequences of glycerol‐3‐phosphate acyltransferase family genes were obtained from NCBI (https://www.ncbi.nlm.nih.gov/) and Gramene (https://ensembl.gramene.org/). Multiple sequence alignment of glycerol‐3‐phosphate acyltransferase family proteins and the construction of neighbour‐joining tree were performed using the MEGA 7.0 software (Kumar et al. 2016).

### 4.7 Recombinant Protein

Recombinant WT and truncated version corresponding to the *Osgpat6.1* mutant protein were generated by amplified their coding sequences via PCR, respectively. Subsequently, the coding sequences of WT and mutant *Osgpat6.1* were cloned into the pGEX‐4 T‐2 vector. The primer sequences used in this study are provided in Table S3. The fusion plasmids were transformed into *Escherichia coli* (*DE3* strain). Protein expression was induced by the addition of 0.5 mM isopropyl β‐D‐thiogalactopyranoside (IPTG) and allowed to proceed overnight at a temperature of 16°C. Then *E. coli* was collected by centrifugation and the pellet was resuspended with the protein buffer consisting 150 mM KCl, 0.2 mM DTT, 20 mM Tris–HCl, pH 8.0. The resuspended bacteria were sonicated to lyse the cells, and the supernatant containing the recombinant protein was isolated by centrifugation. The recombinant protein was collected via magnetic beads (Beaver), and released from the beads using protein eluent at 4°C. The purified proteins were subsequently flash‐frozen in liquid nitrogen and stored at −80°C.

### 4.8 Quantitative Measurement of GAPT Activity

The system to be assayed was added to FA‐SCoA and glycerol‐3‐phosphate (G3P), which produced lysophosphatidic acid (LPA) and CoA‐SH catalysed by glycerol‐3‐phosphate acyltransferase (GPAT). The system was then subjected to the addition of DTNB, which reacted with the CoA‐SH to produce yellow 5‐thio‐2‐nitrobenzoic acid (TNB). The activity of GPAT was then quantified by measuring the TNB absorbance at a wavelength of 412 nm.

### 4.9 The Measurement of Anther Total Lipid Component

More than 60 mg of anthers at stage 12 were selected and added to a centrifuge tube containing 2 mL of 5% hydrochloric acid in methanol, 3 mL of chloroform in methanol (1:1 v/v), and 100 μL of 19‐alkanoic acid methyl ester. The sample was then subjected to heating at 85°C for a duration of 1 h. Subsequent to cooling to room temperature, 1 mL of n‐hexane was added and the sample was extracted by means of shaking for 2 min. Thereafter, the sample was left to stratify for 1 h. The upper layer was extracted with 100 μL of the upper layer solution. 100 μL of the upper layer was taken, fixed to 1 mL, filtered, and analysed by gas chromatography. Three replicates were performed for each genotype.

### 4.10 Observation of Embryo Sac

Observation of embryo sacs was performed as described previously (Zhao et al. 2013). Fresh and mature rice panicles were fixed in Carnoy's solution for at least 24 h at room temperature. The embryo sacs were then released using tweezers and pre‐treated in 70% ethanol for 24 h. They were processed through a series of ethanol concentrations (50%, 30%, and 15%) for 2 h each step, followed by transfer to distilled water. The embryo sacs were stained with 1% eosin‐Y overnight. They were then processed through another series of ethanol concentrations (30%, 50%, 70%, 90%, and 100%) for 2 h each step. Finally, the embryo sacs were soaked in a 1:1 mixture of ethanol and methyl salicylate for 1 h and cleared in 100% methyl salicylate for at least 10 h. The embryo sacs were examined using a Zeiss LSM980 confocal laser scanning microscope with a × 10 objective (0.4 numerical aperture).

### 4.11 Subcellular Localization

Full length coding sequences of *Osgpat6.1* were amplified by PCR and cloned into the pCAMBIA1305‐GFP and pAN580 vectors, respectively. The transient expression in epidermal cells of *N. benthamiana* was performed as described previously (Bai et al. 2019). The pCAMBIA1305‐GFP and pAN580 plasmids were transformed into *N. benthamiana* leaves and rice protoplasts, respectively. GFP and mCherry Fluorescence signals were observed under Zeiss LSM980 laser scanning microscope, which equipped with a × 10 objective (0.4‐numerical aperture). Images were captured using Zen 3.3 software.

### 4.12 Total RNA Extraction and qRT‐PCR

Total RNA was extracted from various rice tissues of the WT using an RNA Prep Pure Plant kit (ZYMO, R1050). Reverse transcription was conducted with 1 μg of total RNA using oligo‐dT, random primers and PrimeScript I (Takara). qRT‐PCR was performed using a SYBR Premix Ex Taq Kit (Takara) on an ABI prism 7500 Real‐Time PCR System (Thermo Fisher Scientific). Gene expression levels were calculated using the 2^−ΔΔCT^ method. The primer sequences used in this study are provided in Table S3.

### 4.13 Accession Number

*OsGPAT6.1* (*LOC_Os10g27330*), *OsGPAT6.2* (*LOC_Os03g52570*), *OsGPAT6.3* (*LOC_Os02g02340*).

## Author Contributions

J.W., Z.Z., and S.Z. supervised the project. Z.Z. and Y.T. provided plant materials. S.C. designed the experiments. S.C. and H.Z. performed data analysis and drafted the manuscript. Y.T., S.L., and X.L. managed plant cultivation. J.L., K.C., X.H., K.S., J.G., B.Y., Y.H., Z.X., L.H., Z.X., Y.Z., S.G., D.L., A.J., X.Z., H.Y., S.Z., M.C., and Y.C. contributed to field phenotyping and sample collection. C.W., X.Y., K.D., J.W., L.J., Y.R., and X.G. provided technical guidance. All authors read and approved the final manuscript.

## Conflicts of Interest

The authors declare no conflicts of interest.

## Supporting information


**Figure S1:** Observations of spikelet, anther and pollen WT and *Osgpat6.1*. (a–h) *Osgpat6.1* showed no difference in spikelet morphology compared to WT (a–d), but the anthers were shorter and slightly paler in colour (e–h). Appearance of anthers (i–l) and pollen grains (m–p) of WT and *Osgpat6.1* under scanning electron microscope (SEM). Scale bars, 1 cm in (a–f), 500 μm in (g, h), 20 μm in (i, j), 5 μm in (k, l), 10 μm in (m, n), 2 μm in (o, p).
**Figure S2:** pbi70649‐sup‐0001‐FiguresS1‐S13.docx. *Osgpat6.1* possesses normal female gametes. (a, b) Observation of mature embryo sacs of WT and *Osgpat6.1*. (c) panicles obtained from pollination of WT by *Osgpat6.1*. (d) Spikelet obtained from pollination of *Osgpat6.1* by WT. (e) Embryo sac fertility statistics of WT and *Osgpat6.1*. (f) Seed setting rate of spikelets obtained by crossing WT and *Osgpat6.1* with each other. Values are means ± SD, *n* = 3. “N.S.” denotes no significant difference, ***p < 0.001 by Student's *t*‐test. Scale bars, 25 μm in (a, b), 5 cm in (c, d).
**Figure S3:** Meiotic chromosome behaviours of WT (a, b) and *Osgpat6.1* (c, d). MetaphaseI (a, c), TelophaseI (b, d). Scale bars, 50 μm.
**Figure S4:** Statistics analysis of phenotypes of plants related to Figure 3.(a–c) Pollen fertility (a), spikelet fertility (b), and plant height (c) of WT and various transgenic lines in NJ4, GLA4, and Kitaake backgrounds under the indicated average temperatures. Values are means ± SD.
**Figure S5:** (a) Analysis of WT and *Osgpat6.1* anther during stage 12 of anther development total lipid component under LT. (b) Relative lipid amounts of *Ospgat6.1* compared to WT under both HT and LT. The dashed horizontal line represents the WT level. Values are means ± SD, *n* = 3 for HT and *n* = 4 for LT. “N.S.” denotes no significant difference, *p < 0.05, **p < 0.01***p < 0.001 by Student's *t*‐test.
**Figure S6:** RNA‐seq analysis and KEGG pathways enriched analysis of the WT and *Osgpat6.1* mutant under HT. (a) Volcano plot showing DEGs between *Osgpat6.1* and WT anthers. log_2_ (FC) indicates fold change, and log_10_ (FPKM) represents expression level. (b) KEGG pathway enrichment analysis of DEGs. The size of each dot represents the number of genes in the pathway, and the colour indicates adjusted *p*‐values.
**Figure S7:** Single and multiple mutants of *OsGPAT6.1* and its homologous genes were generated using CRISPR/Cas9 Genomic structure and the mutation site of *OsGPAT6.1* and its homologous genes. Exons are shown as boxes, and introns as lines. Dash lines represent base deletions, and bold letters represent base insertions.
**Figure S8:** Statistics analysis of phenotypes of plants related to Figure 5.(a–c) Pollen fertility (a), spikelet fertility (b), and plant height (c) of WT, *Osgpat6.2*, *Osgpat6.3*, *cr‐Osgpat6.1#K2*, *Osgpat6.1*#K2 *Ubi::OsGPAT6.2* and *Osgpat6.1*#K2 *Ubi::OsGPAT6.3* under HT. Values are means ± SD. “N.S.” denotes no significant difference, ***p < 0.001 by Student's *t*‐test. (d–f) Pollen fertility (d), spikelet fertility (e), and plant height (f) of WT, *cr‐Osgpat6.1#K2*, *cr‐Osgpat6.1#K2Osgpat6.2*, *cr‐Osgpat6.1#K2Osgpat6.3*, *Osgpat6.2Osgpat6.3* and *cr‐Osgpat6.1#K2Osgpat6.2Osgpat6.3* under LT. Values are means ± SD. Different letters indicate significant differences by ANOVA and Tukey's test, *p* < 0.01.
**Figure S9:** OsGPAT6.1 and its two homologues had highly similar 3D structures predicted by AlphaFold 3. TM, transmembrane domain; AT, acyltransferase domain. The three proteins aligned have highly similar structural features.
**Figure S10:** Subcellular localization of OsGPAT6.1 and its homologues in tobacco leaf cells. HDEL‐mCherry was used as an endoplasmic reticulum (ER) marker. Scale bars, 5 μm.
**Figure S11:** The expression patterns of *OsGPAT6.2* and *OsGPAT6.3* at different stages of anther development were observed by in situ hybridization. Scale bars, 100 μm. Negative control (sense probe) results are shown in Figure 4.
**Figure S12:** Phenotypes of double and triple mutants in the NJ4 background. (a) Schematic of the target location on *OsGPAT6.2* with the editing of *Osgpat6.1Osgpat6.2* and *Osgpat6.1Osgpat6.2 Osgpat6.3*. (b) Schematic of the target location on *OsGPAT6.3* with the editing of *Osgpat6.1Osgpat6.3* and *Osgpat6.1Osgpat6.2Osgpat6.3*. (c) Phenotypic of double mutants *Osgpat6.1Osgpat6.2* and *Osgpat6.1Osgpat6.3*, and triple mutant *Osgpat6.1Osgpat6.2Osgpat6.3* under LT. (d) Statistics of pollen fertility, spikelet fertility, plant height of *Osgpat6.1Osgpat6.2* and *Osgpat6.1Osgpat6.3*, and *Osgpat6.1Osgpat6.2Osgpat6.3* under LT. Different letters indicate significant differences by ANOVA and Tukey's test, *p* < 0.01 gure S13 *Osgpat6.1* is a potentially excellent TGMS gene for Breeding. (a–e) *Osgpat6.1* (NJ4 background) crossed with T65 (a), Dular (b), N22 (c), DJY (d), and NIP (e), espectively (the right of each panel) to generate the F_1_ plants. NJ4 as a control (the left of each panel). Scale bars, 50 cm in the all of the images of plants and 5 cm in all of the images of panicles. (f–i) The plant height (f), number of tiller (g), grain number per panicle (h), and spikelet fertility (i) of the above hybrid combinations. Values are means ± SD, *n* = 10. “N.S.” denotes no significant difference by Student's *t*‐test.

**Table S1:** Genetics analysis of Osgpat6.1 mutant.

**Table S2:** Sequence variations identified in predicted ORFs within the mapped interval.

**Table S3:** Primers in this study.

**Table S4:** Composition of other glycerophospholipids and chloroplast membrane precursor substances in anthers of the during stage 12 in WT and Osgpat6.1 under HT.

**Table S5:** Composition of other glycerophospholipids and chloroplast membrane precursor substances in anthers of the during stage 12 in WT and Osgpat6.1 under LT.

## Data Availability

The data that support the findings of this study are available in the Supporting Information of this article.
