# Supplementary material for: Redundant Glycerol‐3‐Phosphate Acyltransferases Regulate Thermo‐Sensitive Genic Male Sterility in Rice
Source: Plant Biotechnol J. 2026 Apr 1;24(7):4689–703. doi: 10.1111/pbi.70649 (PMC13278554; doi:10.1111/pbi.70649)
Supplement: Supplementary file 1 — Figure S1: Observations of spikelet, anther and pollen WT and Osgpat6.1. (a–h) Osgpat6.1 showed no difference in spikelet morphology compared to WT (a–d), but the anthers were shorter and slightly paler in colour (e–h). Appearance of anthers (i–l) and pollen grains (m–p) of WT and Osgpat6.1 under scanning electron microscope (SEM). Scale bars, 1 cm in (a–f), 500 μm in (g, h), 20 μm in (i, j), 5 μm in (k, l), 10 μm in (m, n), 2 μm in (o, p). Figure S2: pbi70649‐sup‐0001‐FiguresS1‐S13.docx. Osgpat6.1 possesses normal female gametes. (a, b) Observation of mature embryo sacs of WT and Osgpat6.1. (c) panicles obtained from pollination of WT by Osgpat6.1. (d) Spikelet obtained from pollination of Osgpat6.1 by WT. (e) Embryo sac fertility statistics of WT and Osgpat6.1. (f) Seed setting rate of spikelets obtained by crossing WT and Osgpat6.1 with each other. Values are means ± SD, n = 3. “N.S.” denotes no significant difference, ***p < 0.001 by Student's t‐test. Scale bars, 25 μm in (a, b), 5 cm in (c, d). Figure S3: Meiotic chromosome behaviours of WT (a, b) and Osgpat6.1 (c, d). MetaphaseI (a, c), TelophaseI (b, d). Scale bars, 50 μm. Figure S4: Statistics analysis of phenotypes of plants related to Figure 3.(a–c) Pollen fertility (a), spikelet fertility (b), and plant height (c) of WT and various transgenic lines in NJ4, GLA4, and Kitaake backgrounds under the indicated average temperatures. Values are means ± SD. Figure S5: (a) Analysis of WT and Osgpat6.1 anther during stage 12 of anther development total lipid component under LT. (b) Relative lipid amounts of Ospgat6.1 compared to WT under both HT and LT. The dashed horizontal line represents the WT level. Values are means ± SD, n = 3 for HT and n = 4 for LT. “N.S.” denotes no significant difference, *p < 0.05, **p < 0.01***p < 0.001 by Student's t‐test. Figure S6: RNA‐seq analysis and KEGG pathways enriched analysis of the WT and Osgpat6.1 mutant under HT. (a) Volcano plot showing DEGs between Osgpat6.1 and WT anthe [file PBI-24-4689-s004.docx]

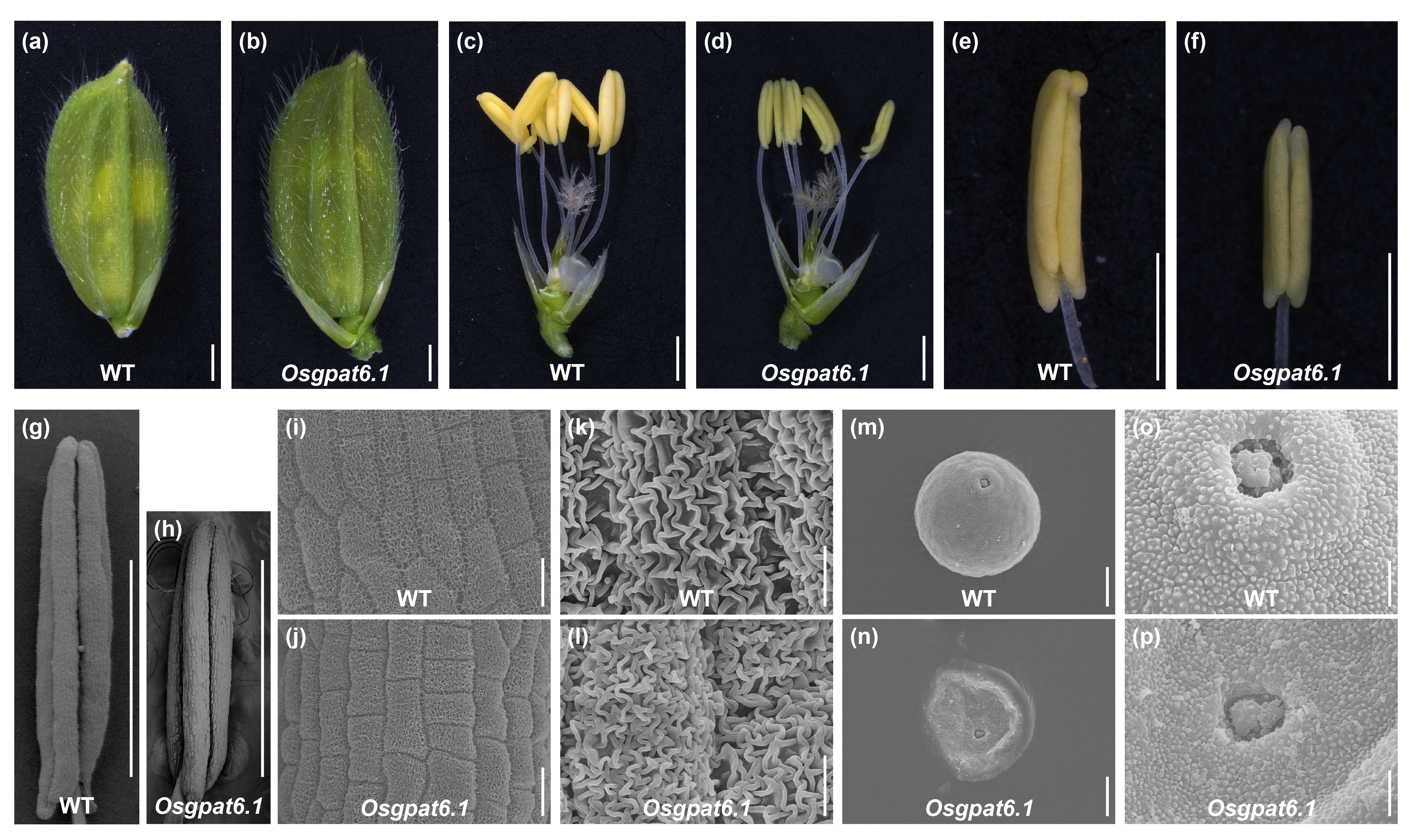


Figure S1 Observations of spikelet, anther and pollen WT and *Osgpat6.1*.

(a-h) *Osgpat6.1* showed no difference in spikelet morphology compared to WT (a-d), but the anthers were shorter and slightly paler in color (e-h). Appearance of anthers (i-l) and pollen grains (m-p) of WT and *Osgpat6.1* under scanning electron microscope (SEM). Scale bars, 1 cm in (a-f), 500 μm in (g, h), 20 μm in (i, j), 5 μm in (k, l), 10 μm in (m, n), 2 μm in (o, p).


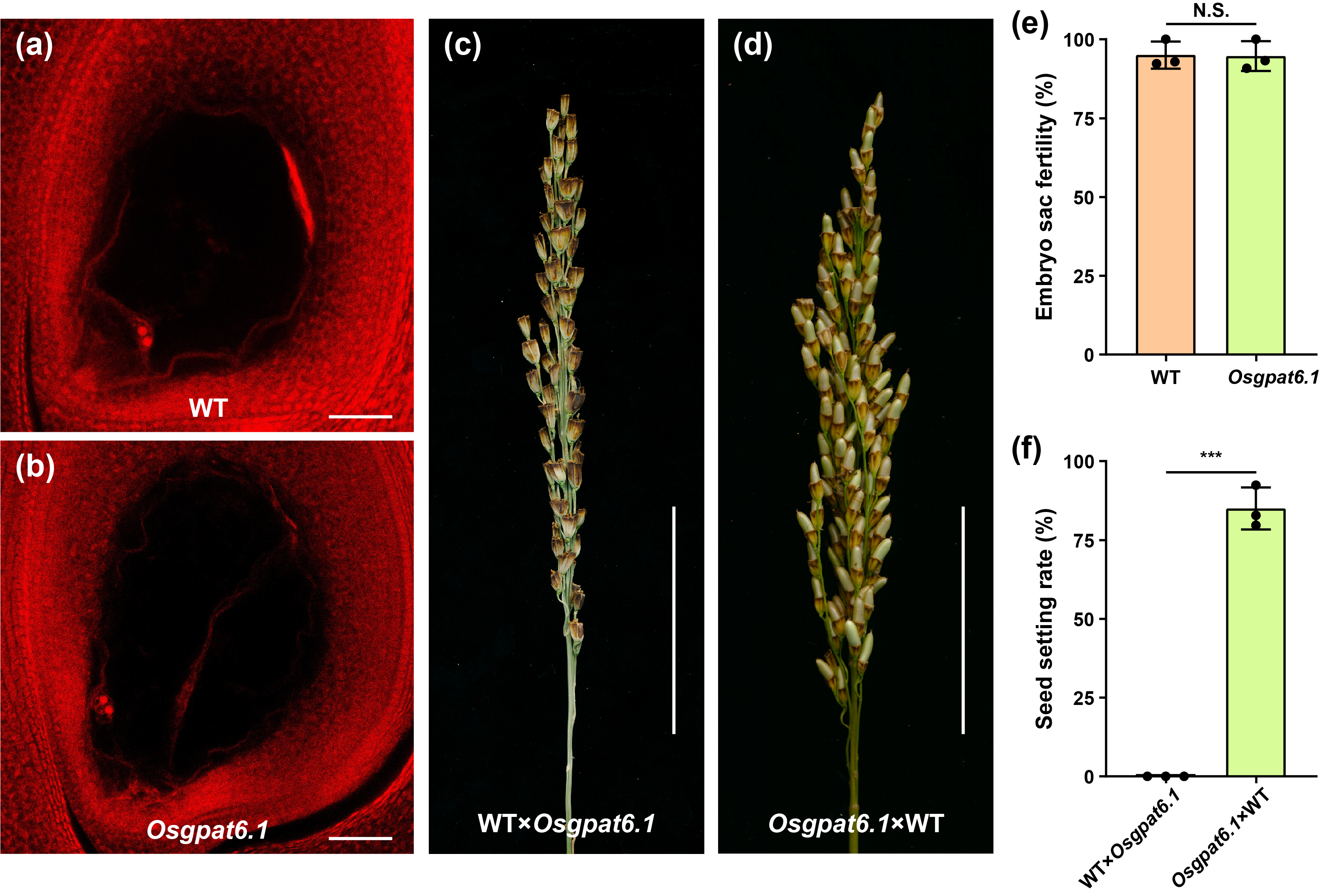


Figure S2 *Osgpat6.1* possesses normal female gametes.

(a, b) Observation of mature embryo sacs of WT and *Osgpat6.1*. (c) panicles obtained from pollination of WT by *Osgpat6.1*. (d) Spikelet obtained from pollination of *Osgpat6.1* by WT. (e) Embryo sac fertility statistics of WT and *Osgpat6.1*. (f) Seed setting rate of spikelets obtained by crossing WT and *Osgpat6.1* with each other. Values are means ± SD, *n* = 3. “N.S.” denotes no significant difference, ****P* < 0.001 by Student’s *t*-test. Scale bars, 25 μm in (a, b), 5 cm in (c, d).


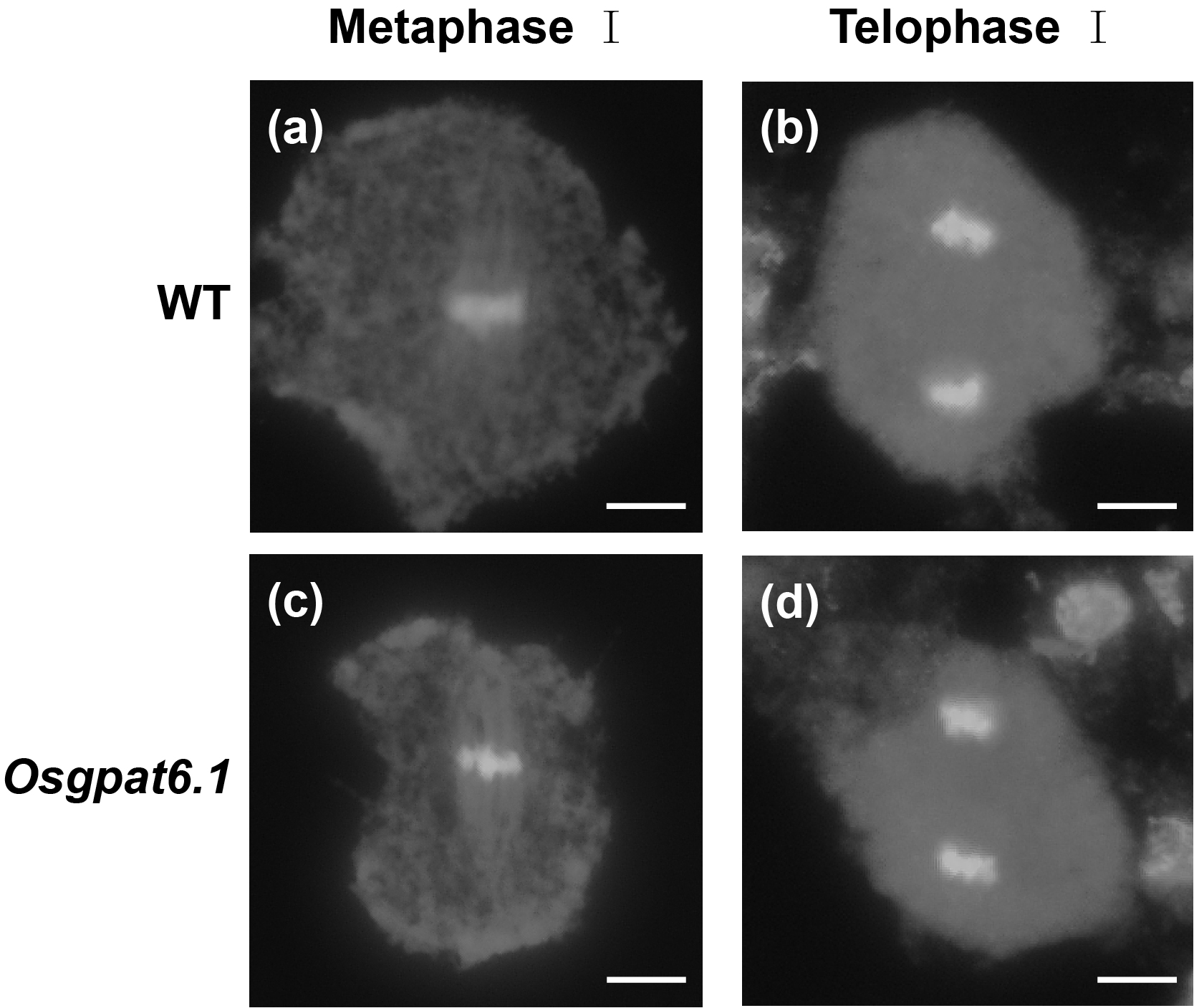


Figure S3 Meiotic chromosome behaviors of WT (a-b) and *Osgpat6.1* (c-d). MetaphaseⅠ (a, c), TelophaseⅠ(b, d). Scale bars, 50 μm.


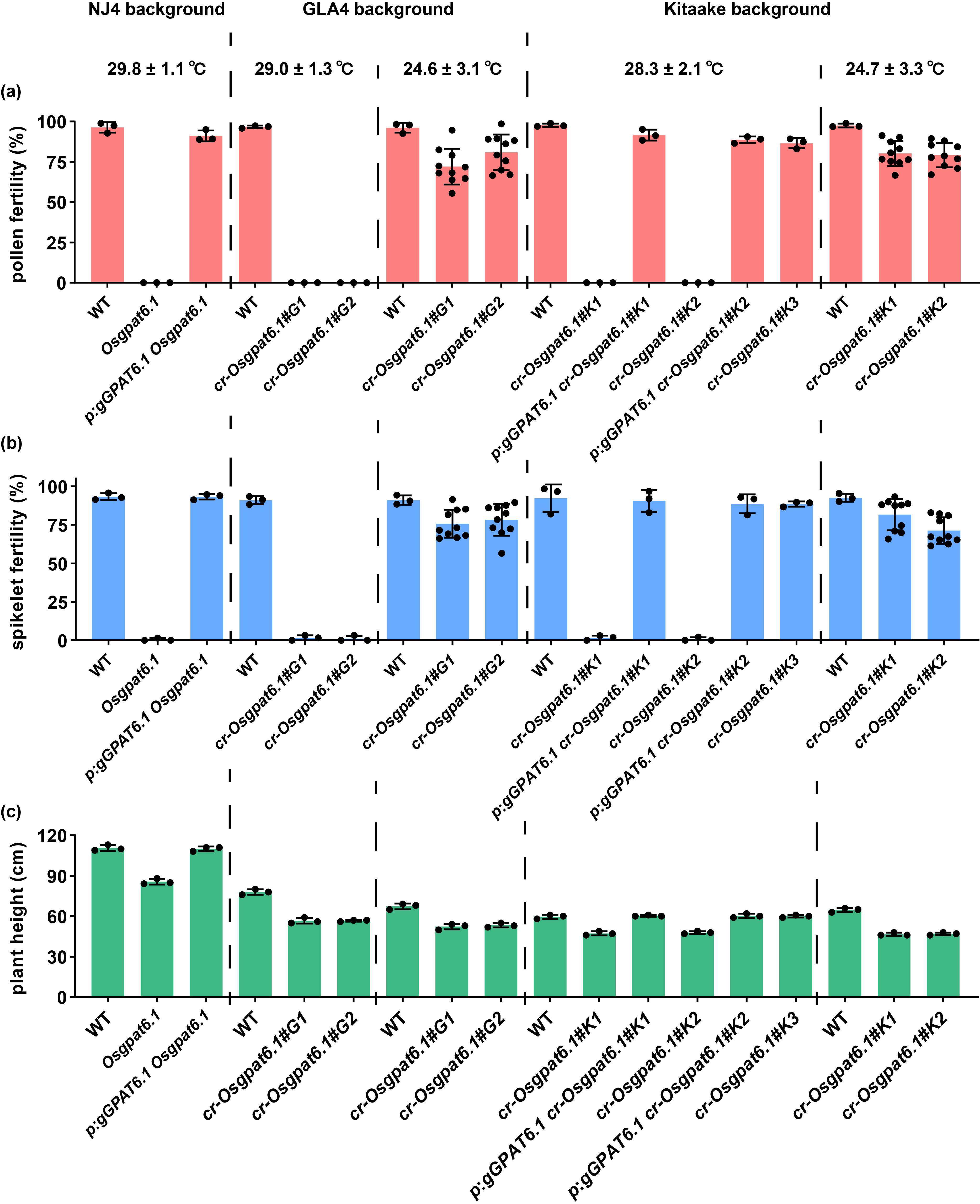


Figure S4 Statistics analysis of phenotypes of plants related to Figure 3.

(a–c) Pollen fertility (a), spikelet fertility (b), and plant height (c) of WT and various transgenic lines in NJ4, GLA4, and Kitaake backgrounds under the indicated average temperatures. Values are means ± SD.


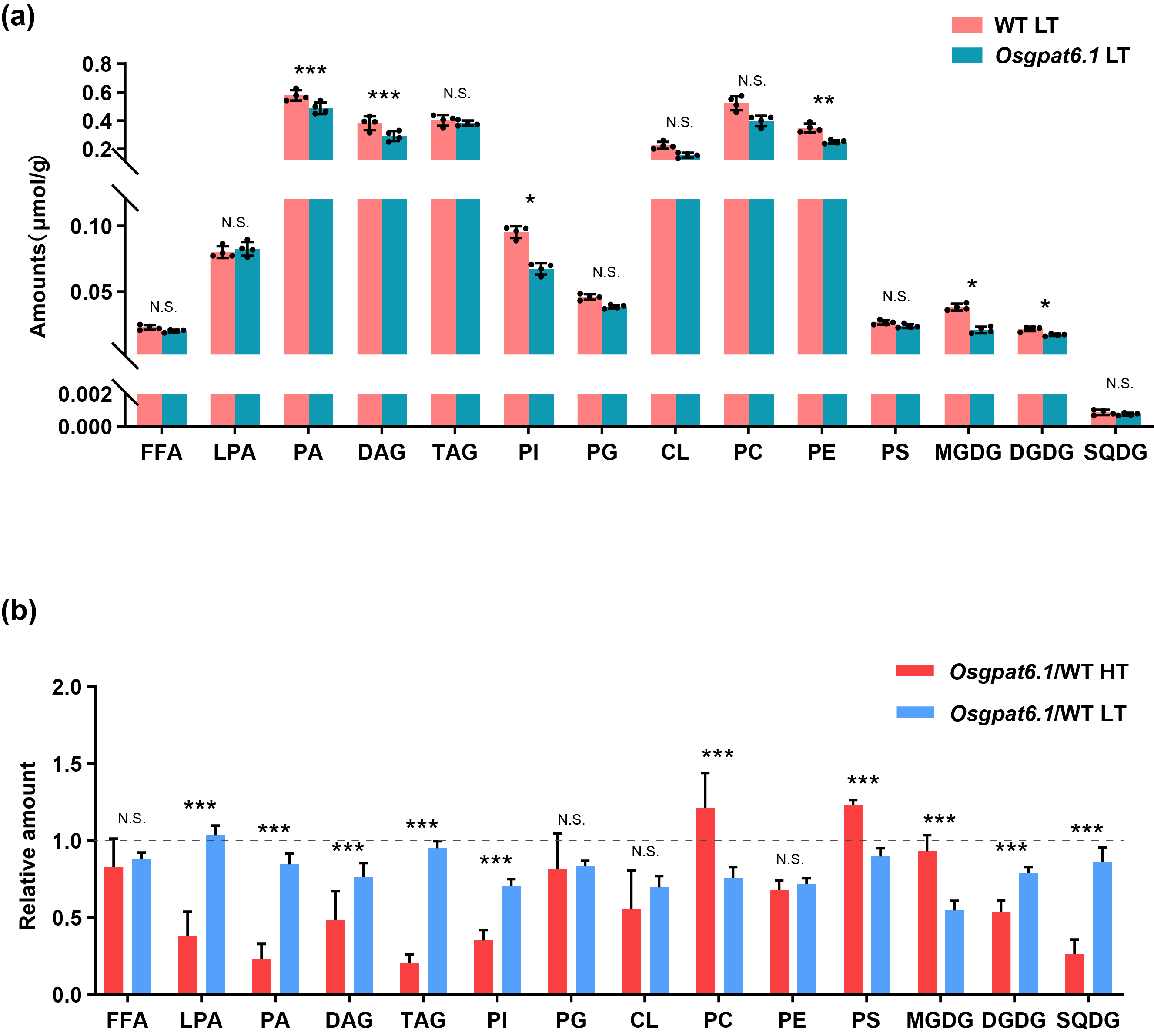


Figure S5 (a) Analysis of WT and *Osgpat6.1* anther during stage 12 of anther development total lipid component under LT. (b) Relative lipid amounts of *Ospgat6.1* compared to WT under both HT and LT. The dashed horizontal line represents the WT level. Values are means ± SD, *n* = 3 for HT and *n* = 4 for LT. “N.S.” denotes no significant difference, **P* < 0.05, ***P* < 0.01****P* < 0.001 by Student’s *t*-test.


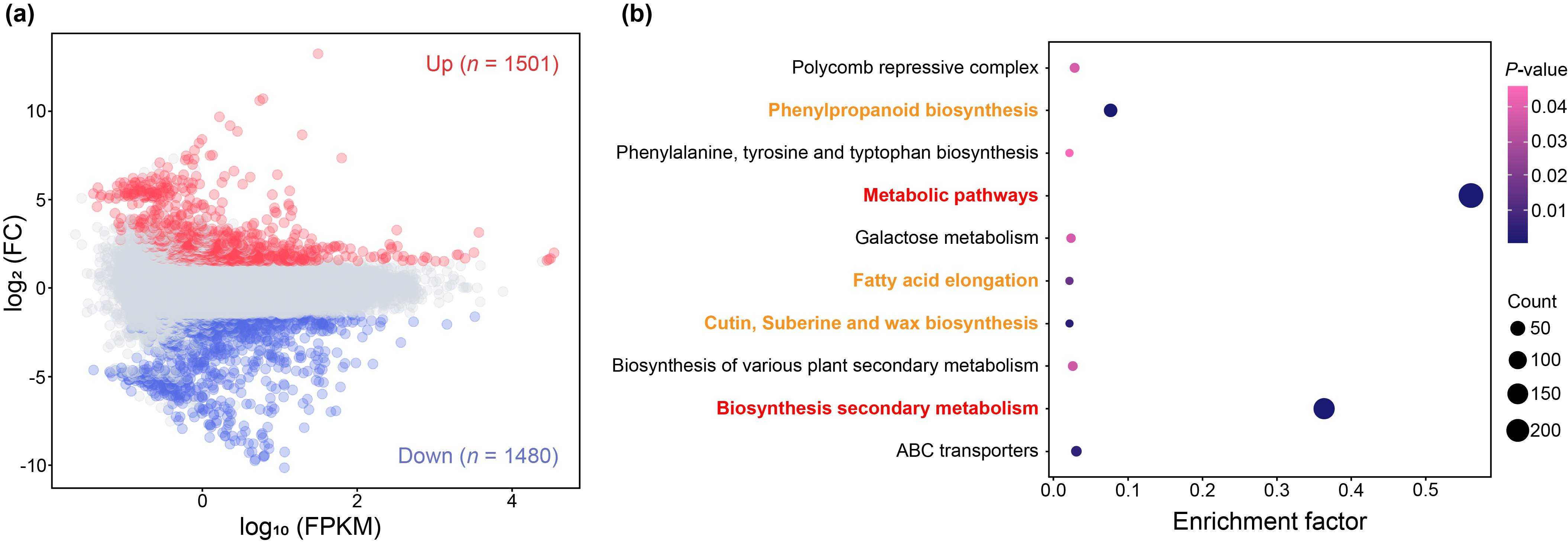


Figure S6 RNA-seq analysis and KEGG pathways enriched analysis of the WT and *Osgpat6.1* mutant under HT.

(a) Volcano plot showing DEGs between *Osgpat6.1* and WT anthers. log_2_ (FC) indicates fold change, and log_10_ (FPKM) represents expression level. (b) KEGG pathway enrichment analysis of DEGs. The size of each dot represents the number of genes in the pathway, and the color indicates adjusted *p*-values.


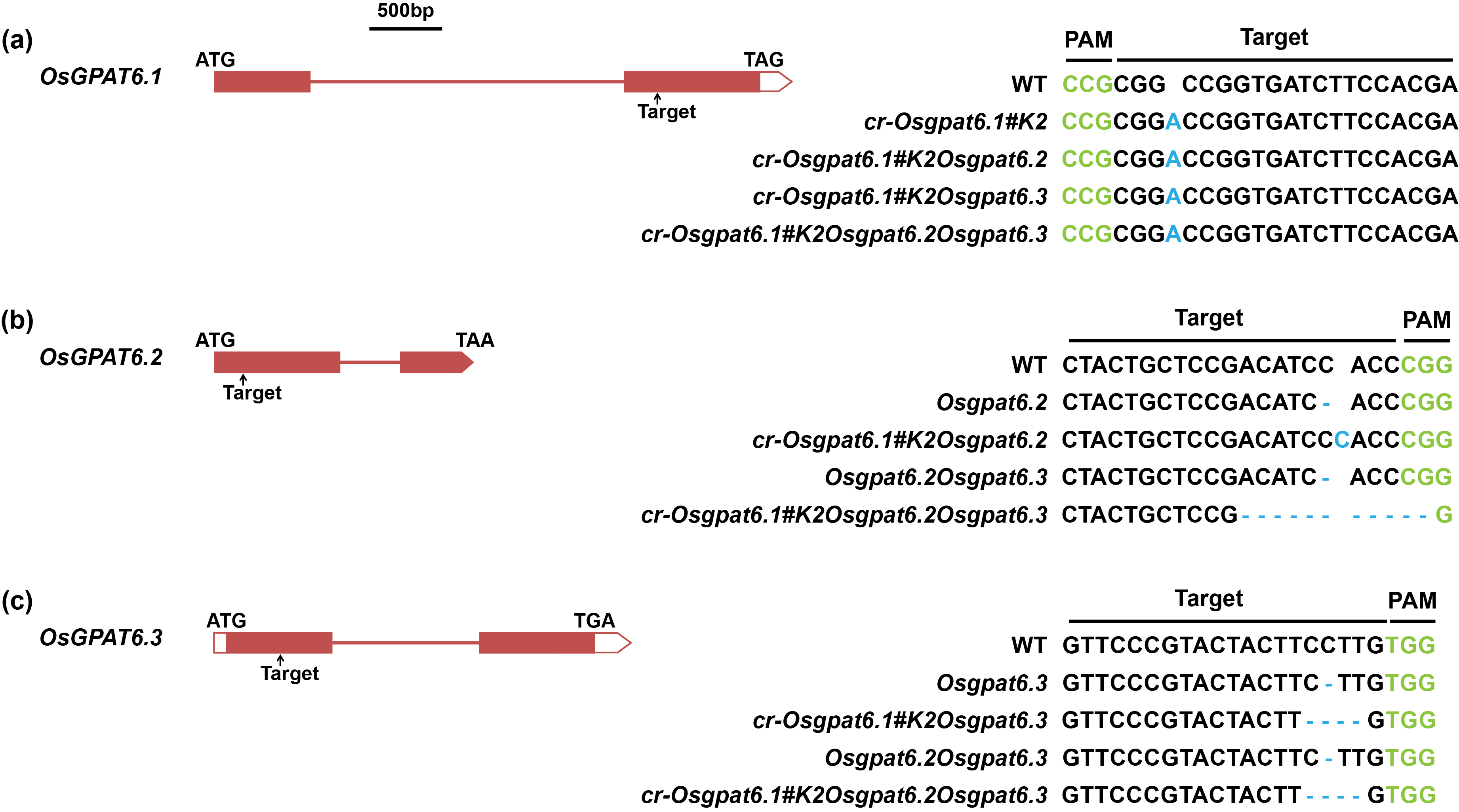


Figure S7 Single and multiple mutants of *OsGPAT6.1* and its homologous genes were generated using CRISPR/Cas9

Genomic structure and the mutation site of *OsGPAT6.1* and its homologous genes. Exons are shown as boxes, and introns as lines. Dash lines represent base deletions, and bold letters represent base insertions


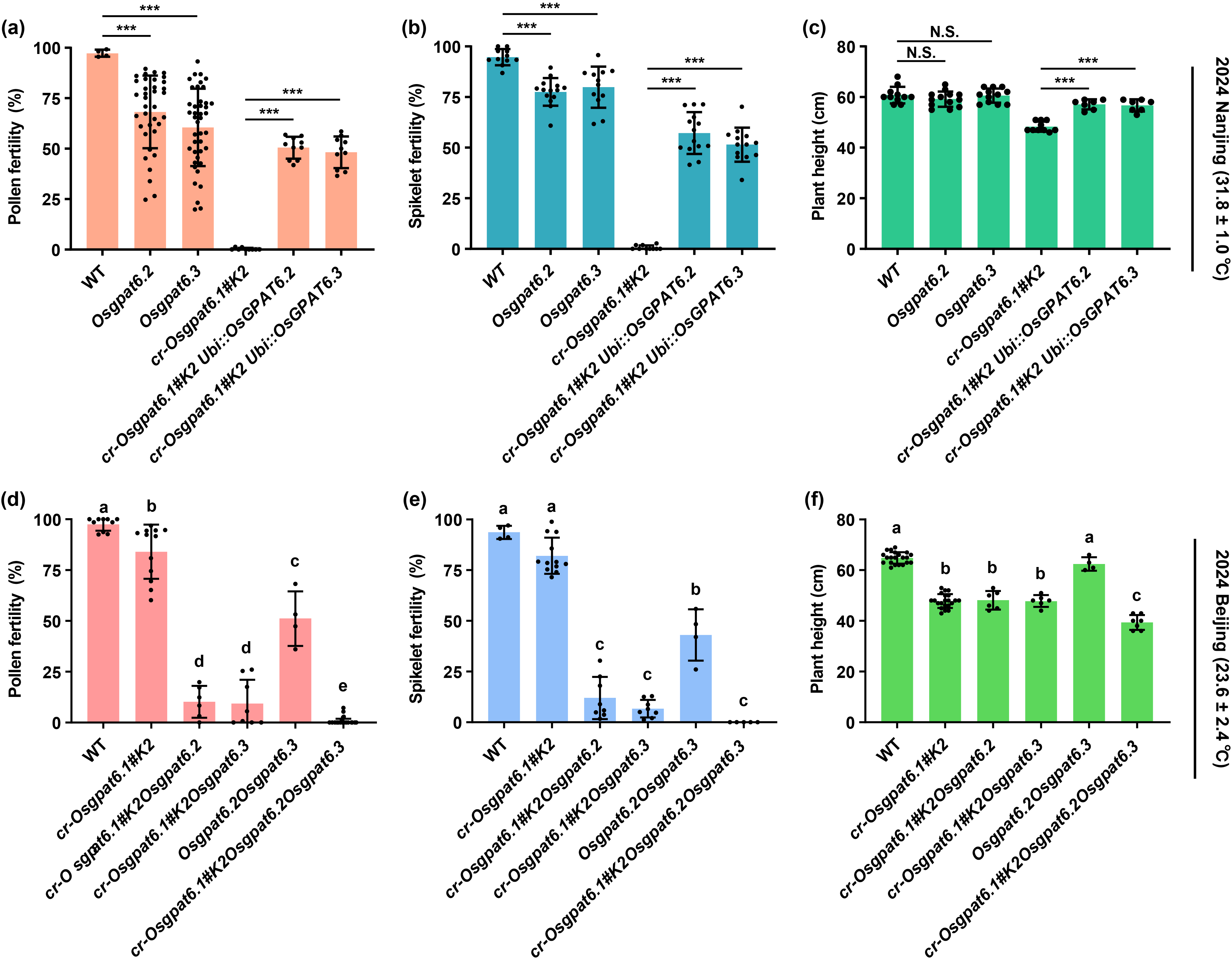


Figure S8 Statistics analysis of phenotypes of plants related to Figure 5.

(a–c) Pollen fertility (a), spikelet fertility (b), and plant height (c) of WT, *Osgpat6.2*, *Osgpat6.3*, *cr-Osgpat6.1#K2*, *Osgpat6.1*#K2 *Ubi::OsGPAT6.2* and *Osgpat6.1*#K2 *Ubi::OsGPAT6.3* under HT. Values are means ± SD. “N.S.” denotes no significant difference, ****P* < 0.001 by Student’s *t*-test. (d–f) Pollen fertility (d), spikelet fertility (e), and plant height (f) of WT, *cr-Osgpat6.1#K2*, *cr-Osgpat6.1#K2Osgpat6.2*, *cr-Osgpat6.1#K2Osgpat6.3*, *Osgpat6.2Osgpat6.3* and *cr-Osgpat6.1#K2Osgpat6.2Osgpat6.3* under LT. Values are means ± SD. Different letters indicate significant differences by ANOVA and Tukey's test, *p* < 0.01.


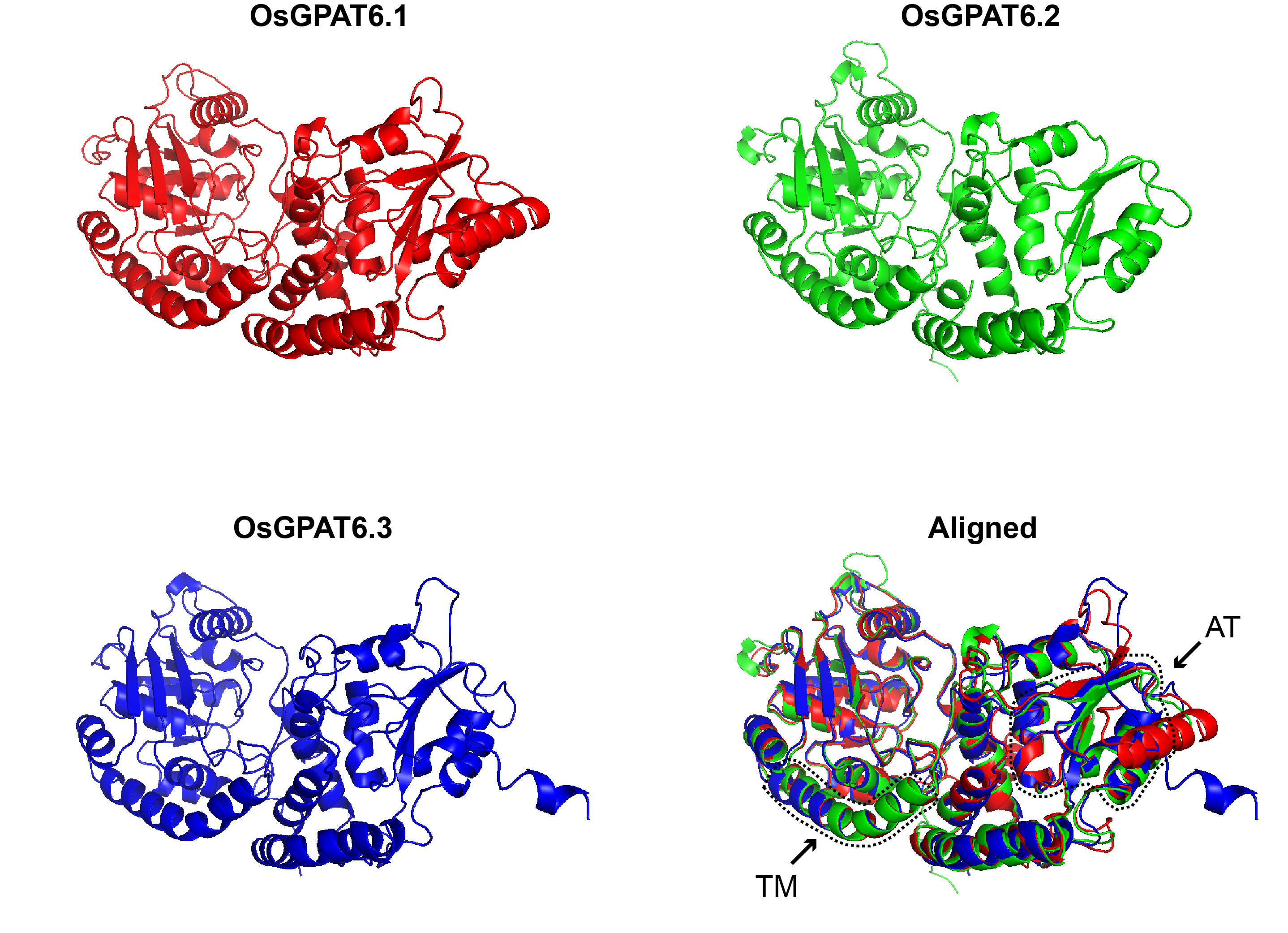


Figure S9 OsGPAT6.1 and its two homologues had highly similar 3D structures predicted by AlphaFold 3. TM, transmembrane domain; AT, acyltransferase domain. The three proteins aligned have highly similar structural features.


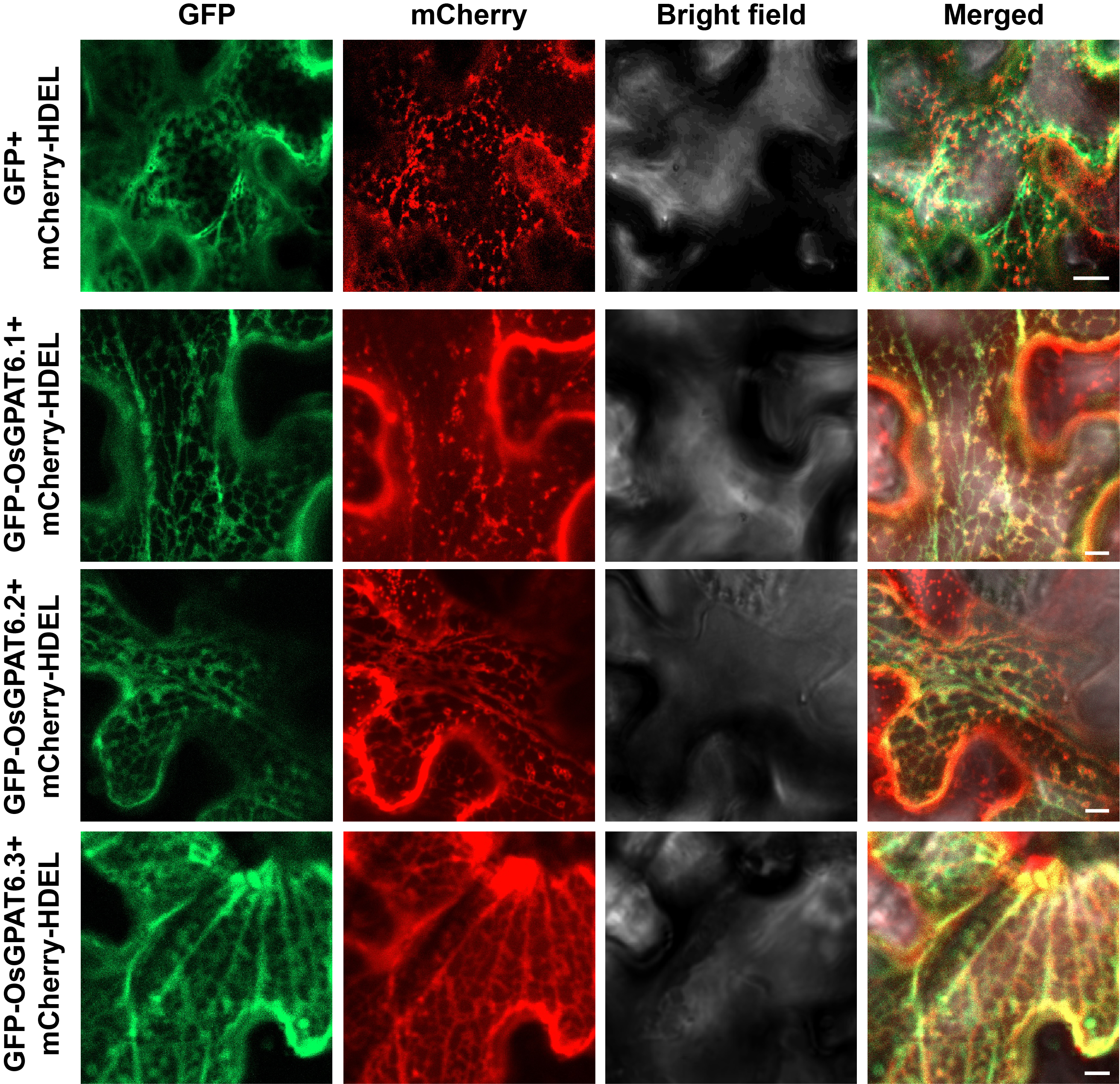


Figure S10 Subcellular localization of OsGPAT6.1 and its homologs in tobacco leaf cells. HDEL-mCherry was used as an endoplasmic reticulum (ER) marker. Scale bars, 5 μm.


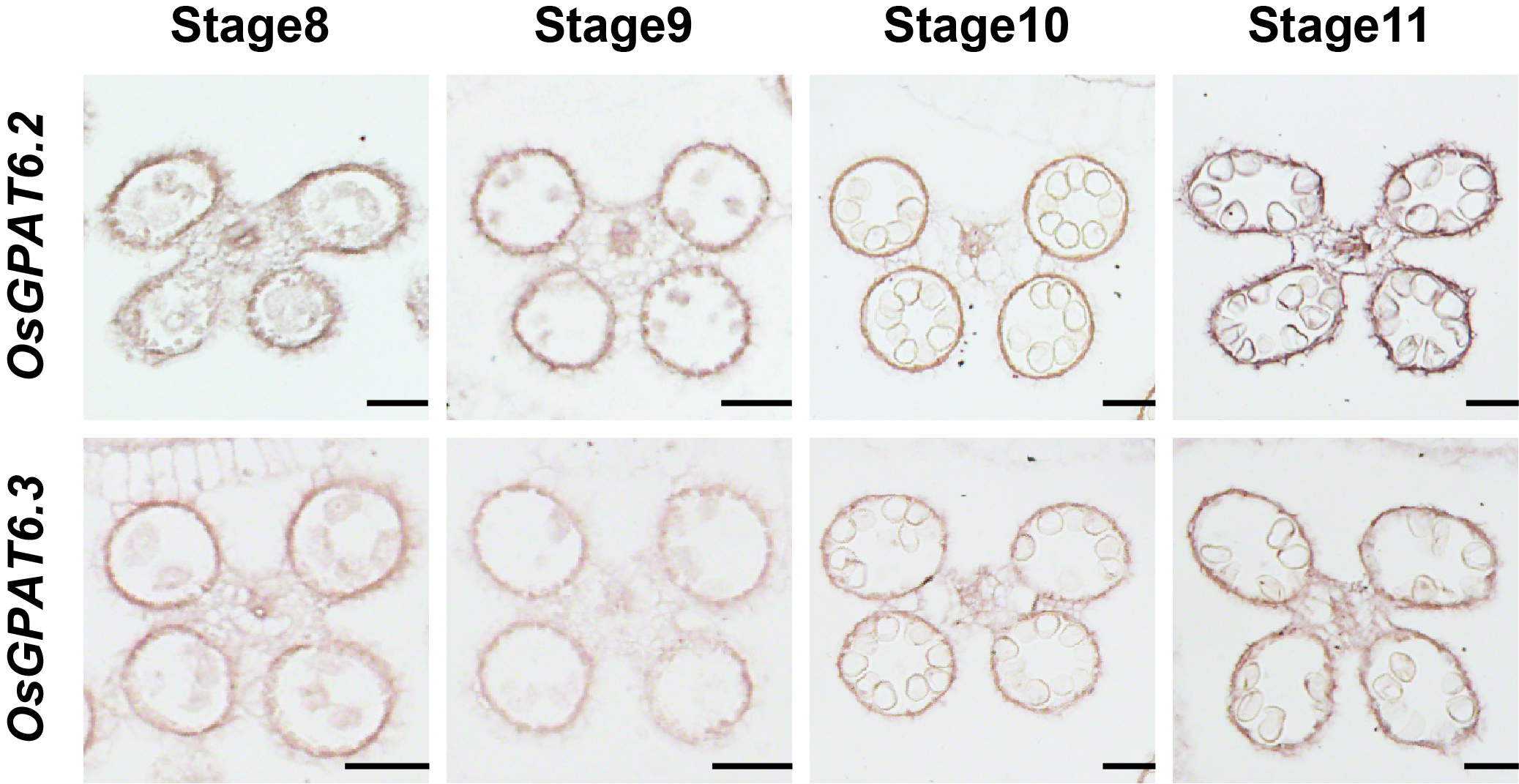


Figure S11 The expression patterns of *OsGPAT6.2* and *OsGPAT6.3* at different stages of anther development were observed by *in situ* hybridization. Scale bars, 100 μm. Negative control (sense probe) results are shown in Figure 4.


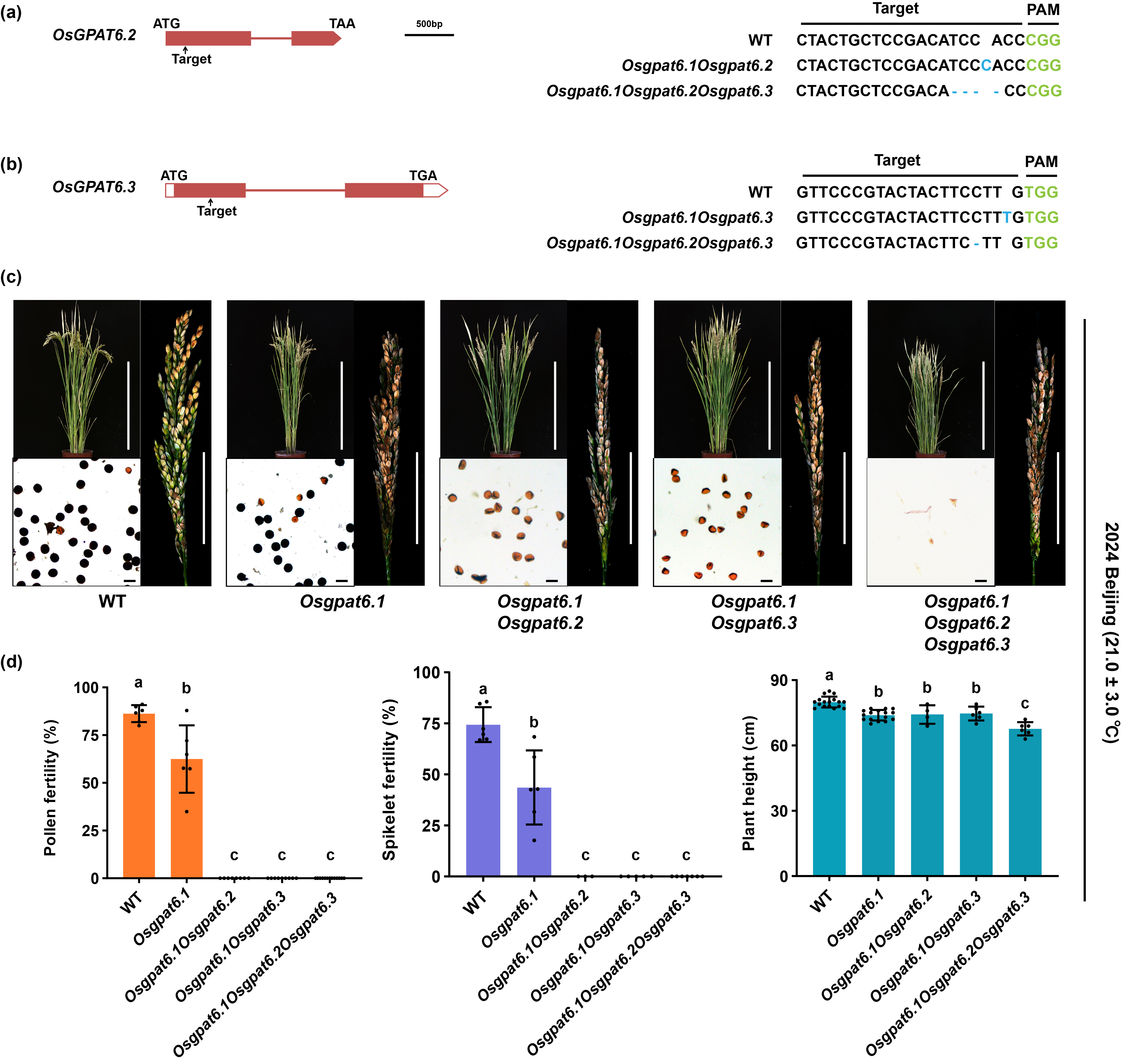


Figure S12 Phenotypes of double and triple mutants in the NJ4 background.

(a) Schematic of the target location on *OsGPAT6.2* with the editing of *Osgpat6.1Osgpat6.2* and *Osgpat6.1Osgpat6.2 Osgpat6.3*. (b) Schematic of the target location on *OsGPAT6.3* with the editing of *Osgpat6.1Osgpat6.3* and *Osgpat6.1Osgpat6.2Osgpat6.3*. (c) Phenotypic of double mutants *Osgpat6.1Osgpat6.2* and *Osgpat6.1Osgpat6.3*, and triple mutant *Osgpat6.1Osgpat6.2Osgpat6.3* under LT. (d) Statistics of pollen fertility, spikelet fertility, plant height of *Osgpat6.1Osgpat6.2* and *Osgpat6.1Osgpat6.3*, and *Osgpat6.1Osgpat6.2Osgpat6.3* under LT. Different letters indicate significant differences by ANOVA and Tukey's test, *p* < 0.01.


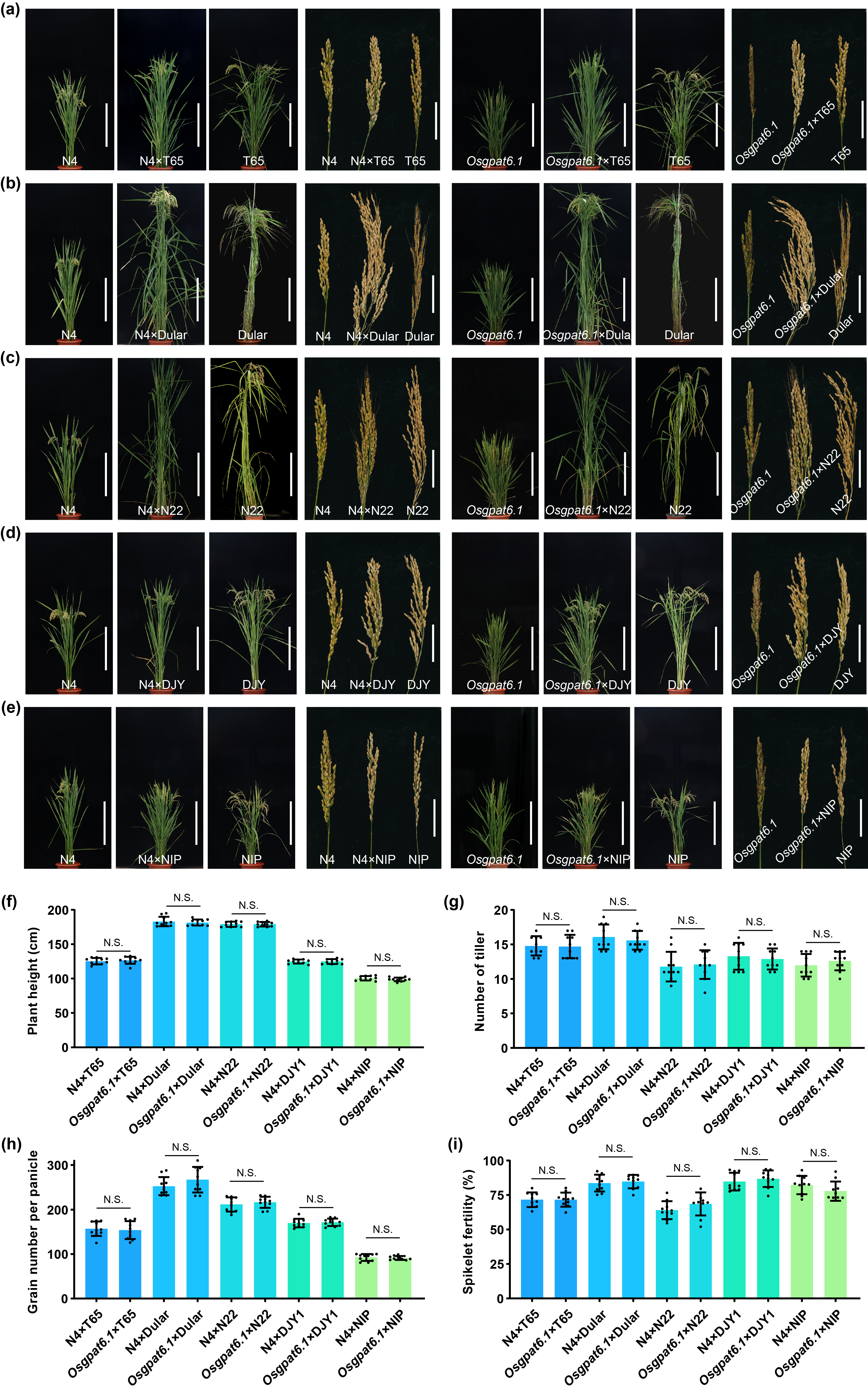


Figure S13 *Osgpat6.1* is a potentially excellent TGMS gene for Breeding.

(a-e) *Osgpat6.1* (NJ4 background) crossed with T65 (a), Dular (b), N22 (c), DJY (d), and NIP (e), respectively (the right of each panel) to generate the F_1_ plants. NJ4 as a control (the left of each panel). Scale bars, 50 cm in the all of the images of plants and 5 cm in all of the images of panicles. (f-i) The plant height (f), number of tiller (g), grain number per panicle (h), and spikelet fertility (i) of the above hybrid combinations. Values are means ± SD, *n* = 10. “N.S.” denotes no significant difference by Student’s *t*-test.
